# Panchromatic Light-Absorbing [70]Fullerene-Perylene-BODIPY Triad with Cascade of Energy Transfer as an Efficient Singlet Oxygen Sensitizer

**DOI:** 10.3390/molecules28083534

**Published:** 2023-04-17

**Authors:** Lifeng Dou, Yuanming Li, Lei Dong, Shuao Zhang, Yuanqi Wu, Yu Gong, Wei Yang, Hongdian Lu, Sane Zhu, Xiaoguo Zhou

**Affiliations:** 1 School of Energy, Materials and Chemical Engineering, Hefei University, Hefei 230601, China; dou_lifeng2010@126.com (L.D.); 15955184794@139.com (L.D.); cloudrrrrrr@126.com (S.Z.); 13329282558@163.com (Y.W.); huayu202035@163.com (Y.G.); weyang@ustc.edu.cn (W.Y.); luhdo@hfuu.edu.cn (H.L.); 2 Hefei National Laboratory for Physical Sciences at the Microscale, Department of Chemical Physics, University of Science and Technology of China, Hefei 230026, China; liym@mail.ustc.edu.cn; 3 State Key Laboratory of Fire Science, University of Science and Technology of China, Hefei 230026, China

**Keywords:** [70]fullerene, perylene, BODIPY, cascade, ping-pong, energy transfer, singlet oxygen

## Abstract

A panchromatic light-absorbing [70]fullerene-perylene-BODIPY triad (**C_70_-P-B**) was synthesized and applied as a heavy atom-free organic triplet photosensitizer for photooxidation. The photophysical processes were comprehensively investigated by the methods of steady-state spectroscopy, time-resolved spectroscopy, as well as theoretical calculations. **C_70_-P-B** shows a strong absorption ability from 300–620 nm. Efficient cascading intramolecular singlet-singlet energy transfer in **C_70_-P-B** was confirmed by the luminescence study. The backward triplet excited state energy transfer from C_70_ moiety to perylene then occurs to populate ^3^perylene*. Thus, the triplet excited states of **C_70_-P-B** are distributed on both C_70_ and perylene moiety with lifetimes of 23 ± 1 μs and 175 ± 17 μs, respectively. **C_70_-P-B** exhibits excellent photooxidation capacity, and its yield of singlet oxygen reaches 0.82. The photooxidation rate constant of **C_70_-P-B** is 3.70 times that of **C_70_-Boc** and 1.58 times that of MB, respectively. The results in this paper are useful for designing efficient heavy atom-free organic triplet photosensitizers for practical application in photovoltaics, photodynamic therapy, etc.

## 1. Introduction

For the wide application of singlet oxygen in the areas of photodynamic therapy (PDT) [1,2,3,4], in vivo oxygen sensing [5], bioimaging [3] and photocatalysis [6,7], researchers have been exploring ways to produce high yields of singlet oxygen in recent years. Singlet oxygen could be generated by photosensitizers (PSs) upon light irradiation in the presence of molecular oxygen. To achieve high photosensitizing efficiency, PSs should exhibit these characteristics, e.g., broadband absorption in the visible or NIR region, high efficiency of intersystem crossing (ISC), long triplet excited state lifetimes, and high photostability [8,9,10]. However, designing PSs with all of the overall properties is a challenge.

The most commonly used strategy for designing triplet PSs is the introduction of heavy atoms [8,11,12,13]. Although heavy-atoms can enhance the ISC to generate triplet states, these PSs still suffer from the weak absorption in the visible range, toxicity, and short triplet excited state lifetimes. A number of other strategies have also been developed to design PSs without heavy atoms, for instance, a twisted π-conjugation system [14,15], radical enhanced intersystem crossing [16], and spin-orbit charge transfer [17,18]. However, there are no clear rules regarding the relationship between the ISC and molecular structure in order to improve singlet oxygen generation efficiency. Moreover, most of the reported PSs are mono-chromophoric, resulting in their narrow absorption in the visible or NIR region. To overcome these shortcomings, connecting multi-chromophores with an electron spin convertor is a good strategy to construct heavy atom free PSs [8]. Benefiting from the high ISC efficiency, unique properties in biological systems and easy functionalization, fullerenes (mainly C_60_) are frequently employed as efficient spin convertors [7,19,20,21,22]. Significantly greater photosensitization efficiency has been achieved in C_60_-based triplet PSs. C_70_, a higher molecular weight fullerene, also possesses high ISC efficiency (near 1.0) [23]. Compared with C_60_, C_70_ has a much more extended system, higher absorption in the visible region, better photodynamic activity, and higher TTA quantum yield [24,25,26]. As a consequence, it is highly promising to synthesize PSs with higher performance using C_70_ as a spin converter. Until now, only a few C_70_-based triplet photosensitizers have been reported [9,27].

**Figure 1:**
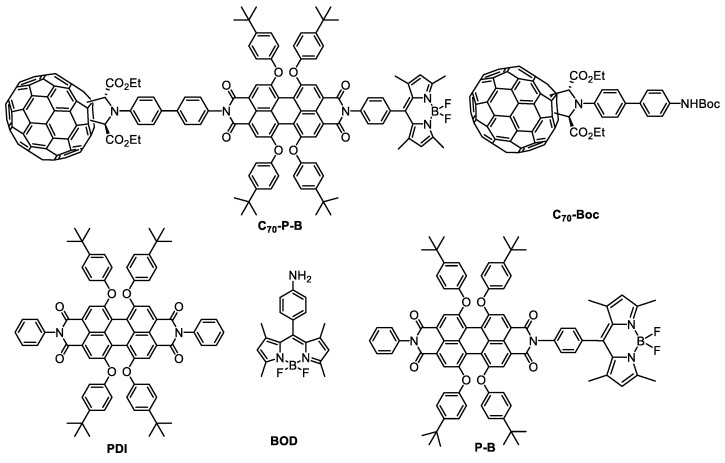
Molecules synthesized and studied in the present work.

In this study, we devised and synthesized a panchromatic light-absorbing [70]fullerene-perylene-BODIPY triad (**C_70_-P-B**) as an efficient singlet oxygen sensitizer using amidation reactions. Considering the strong absorptions in the visible range (at about 505 nm), and energetically high lying excited states, BODIPY was selected as the light harvesting antenna [28]. Perylene, which shows strong absorptions from 525 nm to 620 nm, is complementary in terms of the absorptions to BODIPY. Thus, BODIPY and perylene were selected as light harvesting antennas, and C_70_ was chosen as a spin converter to form a cascade of the energy transfer system. Moreover, the rigid molecular structure can increase ISC rates and quantum yields by suppressing the vibronic deactivation [29]. Thus, BODIPY, perylene and C_70_ were connected through rigid phenyl and biphenyl linkers, respectively. The structure of **C_70_-P-B** is shown in Figure 1. To determine the photophysical properties and photooxidation capacity of **C_70_-P-B**, **C_70_-Boc**, **PDI** [29], **BOD** [30,31] and **P-B** are also prepared as reference compounds. For good solubility, **C_70_-Boc** is synthesized to replace C_70_ as the reference monomer. More stable **PDI** is also synthesized as a reference monomer to replace perylene. The structures of all the reference compounds are also given in Figure 1. As expected, **C_70_-P-B** can efficiently harvest broadband excitation light to generate triplet states. The photophysical processes of **C_70_-P-B** were comprehensively investigated by steady-state UV-visible absorption and fluorescence spectroscopy, time-resolved fluorescence spectroscopy, nanosecond time-resolved transient absorption spectroscopy, as well as theoretical calculations. Thanks to the panchromatic light-absorbing and cascade of energy transfer, **C_70_-P-B** can be used as an efficient photosensitizer to generate singlet oxygen with a high yield. Therefore, our results are very useful for the design and preparation of efficient singlet oxygen sensitizers.

## 2. Results

### 2.1. Synthesis

**Scheme 1:**
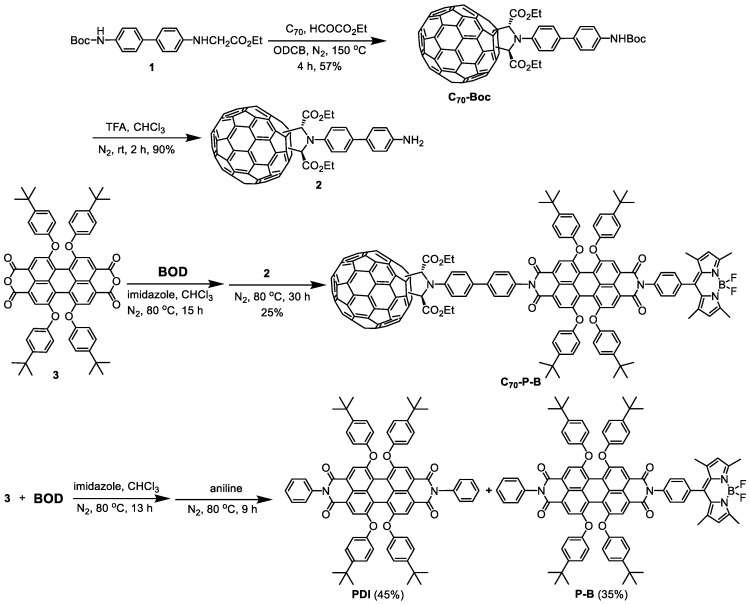
Synthetic procedures for **C_70_-P-B**, the reference compounds **PDI** and **P-B**.

The synthetic routes of the triad **C_70_-P-B** and the reference compounds are shown in Scheme 1, and the details are given in the Section 3.

Compound **1** was synthesized according to the reported procedures of our group [30]. Next, **1** was reacted with glyoxylic acid ethyl ester and C_70_ by 1,3-dipolar cycloaddition in o-dichlorobenzene (ODCB) at 150 °C for 4 h to provide **C_70_-Boc** in 27% yield. Compound **2** could be prepared by removing the Boc-group with TFA. **BOD** was also synthesized following the procedures of our group [31,32]. **BOD** then was reacted with 3,4,9,10-perylenetetracarboxylic dianhydride (**3**) using imidazole as a base to afford the monoanhydride derivative. Monoanhydride has strong adsorption on a silica gel column, and thus it is difficult to get the pure product. Without characterization, the monoanhydride derivative was reacted directly with **2** to synthesize **C_70_-P-B** in 25% yield.

Following the similar procedures, the reference compounds **PDI** and **P-B** were prepared by the cross-condensation of **3**, **BOD** and aniline in 41% and 31% yields, respectively.

**Figure 2:**
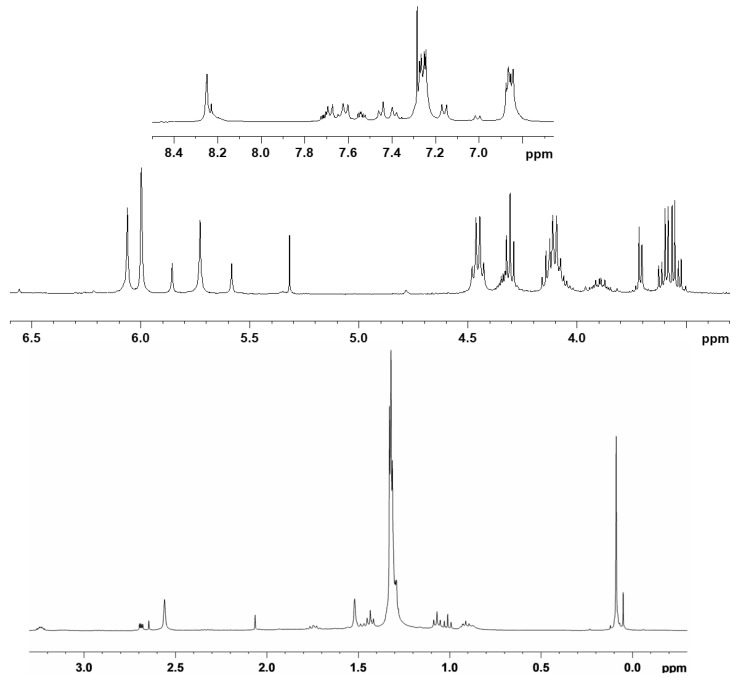
Expansions of ^1^H NMR spectrum of **C_70_-P-B** in CDCl_3_.

The structures of all the new compounds were confirmed by NMR, MS and IR spectroscopy techniques. The NMR and MS spectra of all the compounds are given in the Supplementary Materials. For the sake of clarity, the expansions of the ^1^H-NMR spectrum of **C_70_-P-B** in CDCl_3_ is shown in Figure 2.

The structural symmetry of C_70_ is *D_5h_*, lower than that of C_60_, and, therefore, C_70_ has four different [6,6] ring fusions with different reactivity [33]. As a result, **C_70_-P-B** and **C_70_-Boc** obtained by 1,3-dipolar cycloaddition are a mixture of isomers. From the ^1^H NMR spectrum, it can be concluded that **C_70_-P-B** contains at least three isomers. The peaks at ~8.25–8.19 ppm are assigned to the perylene skeleton protons; peaks at ~7.73–6.84 ppm are assigned to phenyl ring protons; peaks at ~6.01–5.32 ppm are assigned to pyrrolidine protons and pyrrole ring protons; peaks at ~4.48–3.52 ppm are assigned to OC*H*_2_CH_3_; peaks at ~2.64–2.06 ppm are assigned to protons of pyrrole ring C*H*_3_, and peaks at ~1.52–0.87 ppm are assigned to OCH_2_C*H*_3_, protons of the pyrrole ring C*H*_3_ and C(C*H*_3_)_3_ of perylene. The ^13^C NMR spectrum of **C_70_-P-B** also shows the expected signals. For example, the peaks from 169.53–163.01 ppm are assigned to the carbons of *C*=O, the peaks from 157.42–118.97 ppm are assigned to the sp^2^-*C* of C_70_ and the benzene ring, the peaks at ~74.23–72.86 ppm are carbons of *C*HCO_2_, the peaks from 70.88–70.38 ppm are assigned the sp^3^-C of C_70_, peaks from 65.40–60.33 ppm are assigned the carbons of O*C*H_2_CH_3_, peaks from 34.19–29.96 ppm are carbons of *C*(CH_3_)_3_ and C(*C*H_3_)_3_, and peaks from 14.62–13.93 ppm are carbons of *C*H_3_. The mass spectrum of **C_70_-P-B** gives a molecular peak at *m/z* 2482.7023, which is consistent with the calculated data.

### 2.2. UV-Vis Absorption and Steady-State Fluorescence

**Figure 3:**
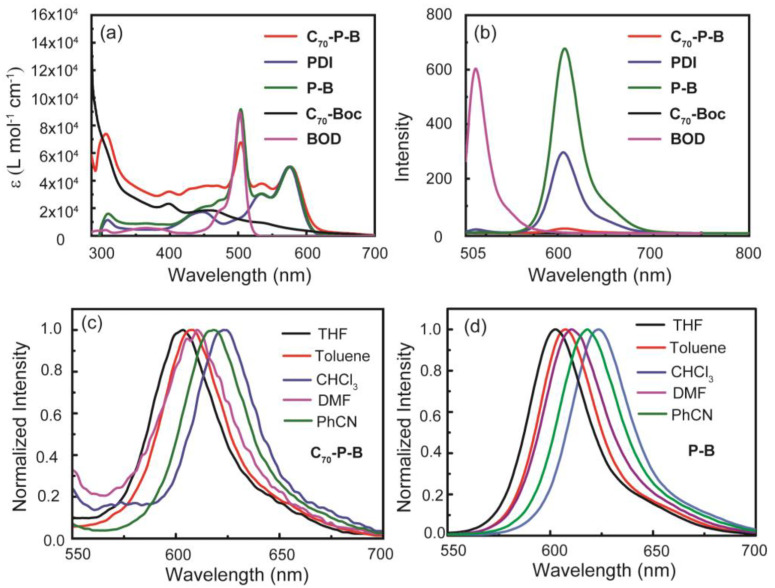
(**a**) UV-vis absorption and (**b**) fluorescence spectra of **C_70_-P-B**, the reference compounds **PDI**, **P-B**, **C_70_-Boc** and **BOD** in toluene (c = 1.0 × 10^−5^ mol/L for **PDI**, **P-B** and **C_70_-Boc**, c = 1.0 × 10^−6^ mol/L for **BOD**). Emission of (**c**) **C_70_-P-B** and (**d**) **P-B** in THF, CHCl_3_, DMF, PhCN and toluene. Exited at 489 nm.

In order to investigate the photophysical properties of the **C_70_-P-B** triad and each component, the UV-vis absorption and steady-state fluorescence spectra of **C_70_-P-B** and the reference compounds were recorded and shown in Figure 3. **C_70_-Boc** with only one C_70_ moiety shows weak absorption in the visible range, and three absorption peaks are located at 398 nm (ε = 23,149 M^−1^ cm^−1^), 465 nm (ε = 17,772 M^−1^ cm^−1^) and 535 nm (ε = 9731 M^−1^ cm^−1^). **P-B** with BODIPY and perylene moieties gives the characteristic absorption peaks of BODIPY at 503 nm and perylene at 575 nm. Intense absorptions at 306 nm (ε = 74,056 M^−1^ cm^−1^) corresponding to the absorption of C_70_, 503 nm (ε = 67,654 M^−1^ cm^−1^) corresponding to the absorption of BODIPY, and 575 nm (ε = 49,166 M^−1^ cm^−1^) corresponding to the absorption of perylene are found for **C_70_-P-B**. Compared with the monomers, the maximum absorption peaks of **C_70_-P-B** and **P-B** are redshifted by about 1 nm. The UV-vis spectrum of **C_70_-P-B** is nearly a superimposition of the monomers, suggesting that electronic communication between **P-B** and C_70_ is weak at the ground state.

The photoinduced intramolecular energy transfer of **C_70_-P-B** and **P-B** was confirmed by the luminescence study. The results are presented in Figure 3b. With photoexcitation at 489 nm, the emission peaks belonging to the C_70_ part in **C_70_-Boc** and **C_70_-P-B** were not detected due to the extremely low fluorescence quantum yield of C_70_ [34,35,36], as well as the overlapping by the fluorescence emission of perylene. In the same conditions, **BOD** gives an intense fluorescence at 514 nm, whereas, due to the efficient intramolecular energy transfer from BODIPY to perylene, this emission is almost completely quenched in both **C_70_-P-B** and **P-B**. **C_70_-P-B**, **P-B** and **PDI** exhibit similar spectral characteristics with an emission peak at 607 nm corresponding to the perylene unit [37,38]. Compared with **PDI**, the luminescence intensity of **P-B** at 607 nm is largely enhanced (the luminescence intensity of **P-B** is 2.27 times that of **PDI**) due to the direct excitation of the perylene moiety and the energy transfer from the BODIPY moiety to perylene upon photoexcitation. Compared with the luminescence intensities of **PDI** and **P-B**, the emission corresponding to perylene moiety is largely quenched in **C_70_-P-B** by the introduction of C_70_. The quenching efficiency of **C_70_-P-B** is about 97% compared to **P-B**. The emission quenching of perylene in **C_70_-P-B** should be ascribed to the efficient excitation energy transfer from perylene to C_70_ upon BODIPY part excitation. The intramolecular energy transfer from the perylene part to C_70_ is thermodynamically allowed, because the energy of the S_1_ state of perylene (2.16 eV) is higher than that of C_70_ (1.85 eV) [9].

To further investigate the effect of solvent polarity on luminescence, the fluorescence emissions of **C_70_-P-B** and **P-B** in THF, CHCl_3_, DMF, PhCN and toluene at the same concentration were measured, as shown in Figure 3c,d. The fluorescence emission intensities are normalized. Clear shifts in emission peaks of **C_70_-P-B** and **P-B** in different solvents could be observed due to the change in dipole moments. Both emission peaks of **C_70_-P-B** and **P-B** are solvent sensitive [30,39].

### 2.3. Time-Resolved Fluorescence Spectroscopy

**Figure 4:**
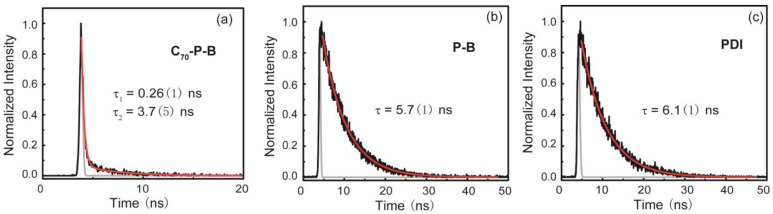
Fluorescence decay traces obtained with a time-correlated single photon counting (TCSPC) of (**a**) **C_70_-P-B**, (**b**) **P-B** and (**c**) **PDI** in toluene. They were excited at 510 nm and recorded at 600 nm. The gray lines are the instrument response profiles, black lines are the raw data, and the red lines are the fitting curves. The brackets represent the error of fitting.

To further study the photo induced intramolecular energy transfer process, the transient fluorescence emission of **C_70_-P-B**, **P-B** and **PDI** were investigated, and a single exponential fit was applied mathematically to all of these compounds. The results are shown in Figure 4.

With photoexcitation at 510 nm, the fluorescence signal at 600 nm was obtained for all three compounds. As shown in Figure 4, all fluorescence decay curves can be perfectly fitted by a single exponential process, and a fast-decay component (gray line, visible in Figure 4a with the full width at half maximum of 0.26 ns), is attributed to the instrument response profile [40]. As a result, the fluorescence lifetimes are determined to be 3.7 ± 0.5 ns for **C_70_-P-B**, 5.7 ± 0.1 ns for **P-B,** and 6.1 ± 0.1 ns for **PDI**, respectively. The l258ifetime of **C_70_-P-B** is apparently shorter than that of **P-B** and **PDI**, and close to the intrinsic lifetime of the C_70_ monomer [36], providing extra evidence for the formation of ^1^C_70_*-P-B by intramolecular energy transfer. **C_70_-P-B** undergoes significant attenuation relative to that of **P-B**, indicating that after the introduction of C_70_, the excited state energy of perylene can be quenched via energy transfer. The results are consistent with that obtained in the steady-state fluorescence spectra. 

### 2.4. Nanosecond Time-Resolved Transient Absorption Spectroscopy

**Figure 5:**
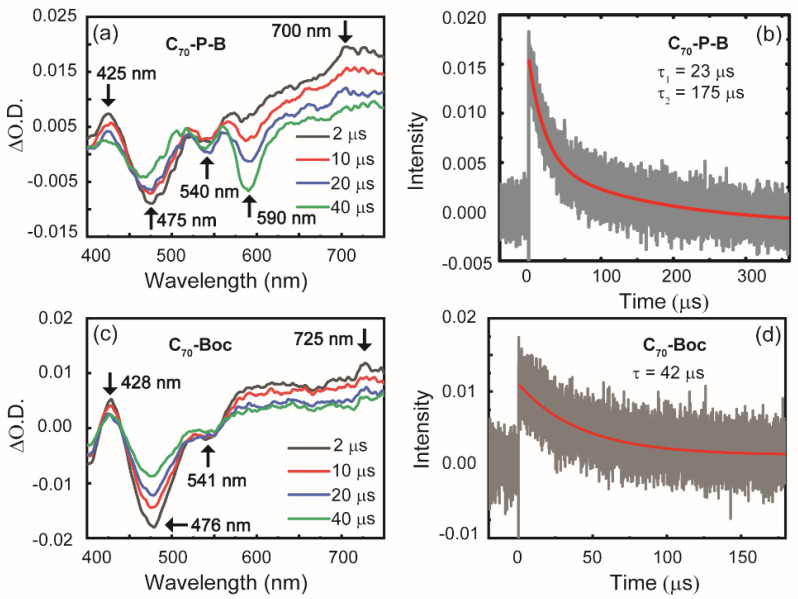
Nanosecond time-resolved transient absorption spectra of (**a**) **C_70_-P-B** and (**c**) **C_70_-Boc** upon excitation at 532 nm with a pulse laser (10 ns, 8 mJ/pulse) in deaerated toluene at room temperature. Decay curves of (**b**) **C_70_-P-B** and (**d**) **C_70_-Boc** at 700 nm. Arrows indicate the spectral trend with time increasing. The gray lines are the raw data, and the red lines are the fitting curves.

To delve into the triplet state properties of **C_70_-P-B** and **C_70_-Boc**, nanosecond time-resolved transient absorption spectra were recorded with photoexcitation at 532 nm, and are displayed in Figure 5a,c, respectively.

For **C_70_-Boc**, a negative peak at 476 nm and two positive absorption bands at 428 nm and ~700 nm were observed. The negative peak could be attributed to the ground-state bleaching (GSB) peak due to its similar position to the absorption of **C_70_-Boc**, while the two positive bands originate from the triplet absorption of the C_70_ moiety. For **C_70_-P-B**, in addition to the three bands at 425 nm, 475 nm and ~700 nm, similar to **C_70_-Boc**, a curiously negative peak at 590 nm was gradually generated. The global fit shows that this negative peak is generated with a characteristic time of 30 ± 2 μs, and is attenuated with a lifetime of 176 ± 11 μs (Figure S1). In comparison with the steady-state absorption spectrum, the negative peak is close to the absorption position of the perylene moiety, and can therefore be attributed to the GSB of the perylene. Considering that the GSB of C_70_ at 475 nm and of perylene at 590 nm shows completely distinct evolution dynamics, it is inferred that there are two different triplet states of this photosensitizer. This deduction could also be verified by the dynamic decay behaviors of **C_70_-P-B** and **C_70_-Boc** at 700 nm. By fitting the kinetic curve of **C_70_-Boc**, the decay of the C_70_ triplet state was identified as mono-exponential, with a lifetime of 42 ± 2 μs. However, the decay process of **C_70_-P-B** was fitted to a bi-exponential form, possessing a short lifetime of 23 ± 1 μs and a longer lifetime of 175 ± 17 μs (Figure 5b). Coincidentally, these two lifetimes matched up with the generation and decay lifetimes of the GSB for perylene. Combined with the result of transient absorption spectra, the shorter lifetime should be ascribed to the triplet state of C_70_; the longer one is the triplet state of perylene. Thus, we propose that the backward triplet energy transfer from C_70_ to the perylene moiety occurs, which also explains why the lifetime of the triplet state of the C_70_ part in the triad is shorter than that in **C_70_-Boc**.

Considering the results of steady-state fluorescence and time-resolved fluorescence spectroscopy parts, a “ping-pong” energy transfer mechanism is proposed for the decay dynamics of **C_70_-P-B** upon photoexcitation. When the BODIPY part is excited, it can transfer energy to the perylene moiety to produce ^1^perylene*. Singlet energy transfer from ^1^perylene* to C_70_ and efficient ISC processes leads to the formation of ^3^C_70_*. According to the optimized geometry of **C_70_-P-B,** as shown in Figure S2, the distance between the BODIPY and perylene units is around 15 Å, and that between the perylene and C_70_ moieties is more than 20 Å. Therefore, Förster energy transfer should dominate for the intramolecular singlet-singlet energy transfer. The backward triplet excited state energy transfer from ^3^C_70_* to perylene then occurs to populate the ^3^perylene*.

### 2.5. TD-DFT Calculations

To validate the energy transfer mechanism mentioned above, density functional theory (DFT) was adapted to calculate the optimized geometry, electronic configuration, vertical excitation energies, and frontier molecular orbitals of **C_70_-P-B**. The optimized structure of **C_70_-P-B** is shown in Figure S2, and the computational details are also available in the Supplementary Materials section.

**Figure 6:**
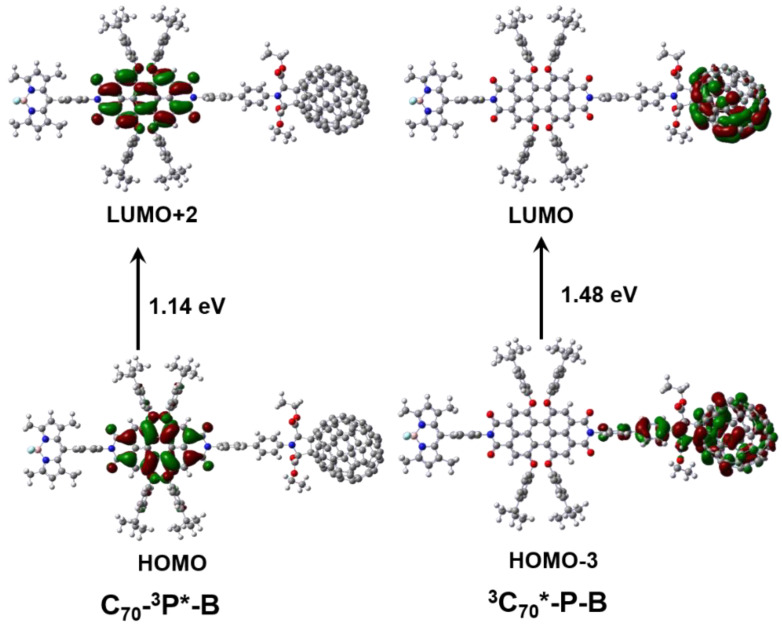
Frontier molecular orbitals involved in the triplet excited states of the **C_70_-P-B**, in which the vertical excitation energy is labelled. The calculations were performed at the TD-DFT//B3LYP/6-31G(d) based on the DFT//B3LYP/6-31G(d) optimized ground state geometries using toluene as the solvent.

Figure 6 shows the transition features of the two lowest triplet states, which are attributed to the HOMO→LUMO+2 and HOMO-3→LUMO transitions, respectively. Moreover, HOMO and LUMO+2 are primarily distributed on the perylene group, whereas HOMO-3 and LUMO are mostly located on the C_70_ moiety. Consequently, these two transitions are the local excitation for perylene and C_70_, respectively, hence the designations C_70_-^3^P*-B and ^3^C_70_*-P-B. Moreover, as shown in Figure 6, the excitation energies of ^3^C_70_*-P-B and C_70_-^3^P*-B are calculated to be 1.48 and 1.14 eV, respectively. The slightly high energy of ^3^C_70_*-P-B confirms that the triplet energy transfer from the C_70_ moiety to the perylene unit in the triad is thermodynamically feasible, which is in line with our experimental conclusions.

### 2.6. Photooxidation of 1,5-Dihydroxy Naphthalene Mediated by ^1^O_2_

Using **C_70_-P-B**, **C_70_-Boc** and **P-B** as singlet oxygen sensitizers, their photooxidation properties were explored by using DHN as a chemical sensor and MB as a reference.

**Figure 7:**
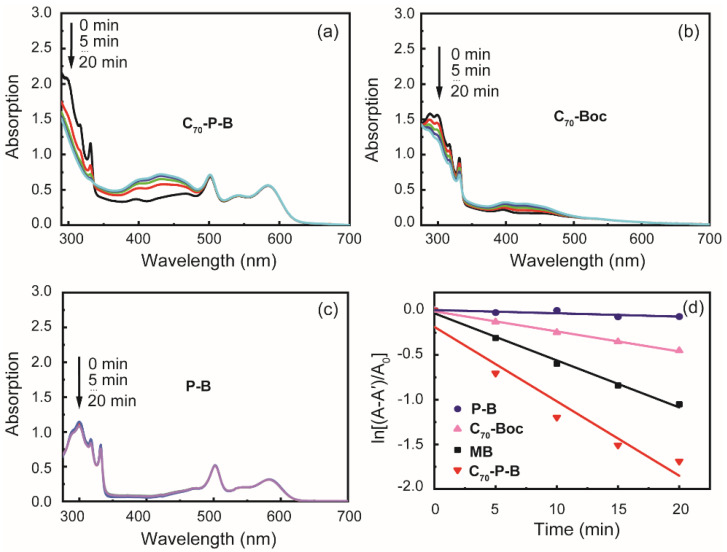
Absorption spectral evolution for the photooxidation of DHN using (**a**) **C_70_-P-B**, (**b**) **C_70_-Boc** and (**c**) **P-B** as photosensitizers. (**d**) Plots of ln[(A − A′)/A_0_ ] vs. irradiation time (t) for the photooxidation of DHN using different sensitizers (collected at 301 nm). c[sensitizers] = 1.0 × 10^−5^ mol L^−1^, c[DHN] = 1.0 × 10^−4^ mol L^−1^. In CH_2_Cl_2_/MeOH (9:1, *v*/*v*). A and A_0_ were the absorbances at 301 nm, where A was the absorption of DHN and sensitizer, A_0_ was the absorption of sensitizer, and A′ was the initial absorption of DHN.

In the presence of ^1^O_2_, DHN can be easily oxidized to juglone. The photooxidation kinetics can be measured by following the decrease in the absorption of DHN at 301 nm or the increase in the absorption of juglone at 427 nm with time. The spectral responses of DHN using **C_70_-P-B**, **C_70_-Boc**, **P-B** and MB as the sensitizers upon excitation with a xenon lamp are presented in Figure 7 and Figure S3, respectively. For **C_70_-P-B**, **C_70_-Boc** and MB, the change of the absorption at 301 nm is obvious, indicating the significant consumption of DHN and the efficient photosensitization ability of the triplet PSs, whereas, nearly no UV-vis absorption change is observed in the spectral responses of DHN with **P-B** as the photosensitizer. The photostability of **C_70_-P-B** was also investigated by exposing it to light for 1 h, and no decrease was observed in the absorption (Figure S4). This further proves that the decrease in the absorption at 301 nm is caused by photooxidation instead of the decomposition of the photosensitizers.

The photooxidation ability of the triplet photosensitizers was quantitatively compared by plotting the ln[(A − A′)/A_0_] against the irradiation time. The photooxidation rate constant and the yield of singlet oxygen (Φ_∆_), together with other photophysical properties of the photosensitizers, were calculated and are listed in Table 1.

## 3. Materials and Methods

### 3.1. Materials

All reagents were obtained from commercial sources. The C_70_, 1,6,7,12-tetra-tert-butylphenoxyperylene-3,4,9,10-tetracarboxylic dianhydride, ethyl glyoxylate, chloroform-*d* (CDCl_3_), trifluoroacetic acid (TFA), anhydrous sodium sulphate (Na_2_SO_4_) and DHN were purchased from Alfa Aesar. The imidazole, aniline, and 1,2-dichlorobenzene (ODCB) were purchased from J&K Scientific Ltd. (Beijing, China). The silica gel, carbon disulfide (CS_2_), dichloromethane (DCM), petroleum ether (PE), methanol (MeOH), tetrahydrofuran (THF), trichloromethane (CHCl_3_), and toluene were purchased from Sinopharm Chemical Reagent Co., Ltd. (Shanghai, China). THF was distilled over the sodium and benzophenone, while other reagents used for the synthesis were used directly.

All compounds were characterized by ^1^H and ^13^C NMR spectroscopy on a BRUKER 400 MHz spectrometer. The mass analyses were performed using a Bruker ultrafleXtreme MALDI TOF/TOF (Bremen, Germany).

### 3.2. Synthesis

#### 3.2.1. Synthesis of **C_70_-Boc**

C_70_ (200.0 mg, 0.24 mmol), compound **1** [30] (180.0 mg, 0.48 mmol) and ODCB (8 mL) were added into a 25 mL, three-neck flask equipped with a gas inlet adaptor. The mixture was stirred at room temperature under an N_2_ atmosphere for 1 h. The ethyl glyoxylate solution (550 µL, 2.8 mmol) was added immediately, and the mixture was stirred at 150 °C for 4 h. The solvent was then removed, and the mixture was subjected to column chromatography on silica gel with CS_2_/CH_2_Cl_2_ (3:2) as eluent to give **C_70_-Boc** as a brown orange powder (83.9 mg, 27%). ^1^H NMR (400 MHz, CDCl_3_) δ 7.49 (d, *J* = 8.7 Hz, phenyl ring H), 7.45–7.43 (m, phenyl ring H), 7.41–7.34 (m, phenyl ring H), 7.07 (d, *J* = 8.7 Hz, phenyl ring H), 6.91 (d, *J* = 8.8 Hz, phenyl ring H), 6.41 (bs, N*H*Boc), 6.39 (bs, N*H*Boc), 6.00 (s, C*H*CO_2_), 5.78 (s, C*H*CO_2_), 5.67 (s, C*H*CO_2_), 5.50 (s, C*H*CO_2_), 4.40 (q, *J* = 7.1 Hz, OC*H*_2_CH_3_), 4.31–4.23 (m, OC*H*_2_CH_3_), 4.10–4.02 (m, OC*H*_2_CH_3_), 3.88–3.78 (m, OC*H*_2_CH_3_), 1.52 (s, OC(C*H*_3_)_3_), 1.51 (s, OC(C*H*_3_)_3_), 1.40 (t, *J* = 7.1 Hz, OCH_2_C*H*_3_), 1.29 (t, *J* = 7.1 Hz, OCH_2_C*H*_3_), 1.04 (t, *J* = 7.1 Hz, OCH_2_C*H*_3_), 0.87 (t, *J* = 7.1 Hz, OCH_2_C*H*_3_). ^13^C NMR (100 MHz, CS_2_/CDCl_3_ with Cr(acac)_3_ as relaxation reagent) δ 170.05, 169.99, 169.47, 157.63, 155.31, 155.11, 154.62, 152.76, 151.53, 151.46, 151.23, 151.18, 150.92, 150.83, 150.80, 150.61, 150.50, 150.38, 150.00, 149.97, 149.85, 149.43, 149.32, 149.22, 149.18, 148.94, 148.83, 148.80, 147.56, 147.53, 147.29, 147.25, 147.16, 147.06, 147.01, 146.92, 146.31, 146.26, 146.20, 146.11, 146.05, 145.95, 144.55, 143.72, 143.58, 143.55, 143.51, 143.40, 143.28, 143.24, 141.09, 140.45, 140.41, 140.17, 138.20, 137.48, 137.44, 135.27, 135.23, 134.65, 133.89, 133.75, 133.17, 132.50, 132.25, 131.93, 131.72, 131.52, 131.49, 131.33, 127.71, 127.58, 127.29, 127.23, 119.05, 118.90, 118.85, 80.68,74.48, 73.20, 72.86, 70.70, 64.82, 63.98, 62.30, 62.08, 61.90, 61.70, 61.68, 29.89, 28.48, 14.65, 14.52, 14.33, 14.13. FT-IR ν/cm^−1^ (KBr) 2922, 2851, 1731, 1611, 1504, 1428, 1401, 1367, 1260, 1158, 1021, 818, 796, 534. HRMS (MALDI-TOF) *m*/*z* calcd for C_95_H_30_N_2_O_6_ [M^−^.] 1294.2109, found 1294.2104.

#### 3.2.2. Synthesis of Compound **2**

**C_70_-Boc** (100.0 mg, 0.08 mmol) was dissolved in chloroform (10 mL). The mixture was stirred under an N_2_ atmosphere at room temperature for 10 min. TFA (2 mL) was then added, and the mixture was stirred at room temperature for 1.5 h until **C_70_-Boc** disappeared. The mixture was purified on a silica gel column using CS_2_/CH_2_Cl_2_ (1:4) as eluent to give compound **2** as a brown orange powder (87.7 mg, 95%). ^1^H NMR (400 MHz, CDCl_3_) δ 7.46 (d, *J* = 8.6 Hz, phenyl ring H), 7.35 (d, *J* = 8.7 Hz, phenyl ring H), 7.31 (d, *J* = 8.4 Hz, phenyl ring H), 7.25 (d, *J* = 8.5 Hz, phenyl ring H), 7.05 (d, *J* = 8.6 Hz, phenyl ring H), 6.89 (d, *J* = 8.6 Hz, phenyl ring H), 6.66 (d, *J* = 8.4 Hz, phenyl ring H), 6.62 (d, *J* = 8.5 Hz, phenyl ring H), 5.99 (s, C*H*CO_2_), 5.78 (s, C*H*CO_2_), 5.66 (s, C*H*CO_2_), 5.50 (s, C*H*CO_2_), 4.40 (q, *J* = 7.1 Hz, OC*H*_2_CH_3_), 4.30–4.22 (m, OC*H*_2_CH_3_), 4.05 (q, *J* = 8.0 Hz, OC*H*_2_CH_3_), 3.89–3.78 (m, OC*H*_2_CH_3_), 3.64 (bs, N*H*_2_), 1.39 (t, *J* = 7.1 Hz, OCH_2_C*H*_3_), 1.28 (t, *J* = 7.1 Hz, OCH_2_C*H*_3_), 1.04 (t, *J* = 7.1 Hz, OCH_2_C*H*_3_), 0.96 (t, *J* = 7.5 Hz, OCH_2_C*H*_3_), 0.86 (t, *J* = 7.1 Hz, OCH_2_C*H*_3_). ^13^C NMR (100 MHz, CS_2_/CDCl_3_ with Cr(acac)_3_ as relaxation reagent) δ 169.72, 169.69, 169.66, 157.53, 154.97, 154.53, 151.40, 151.32, 151.10, 151.05, 150.82, 150.67, 150.50, 150.38, 150.28, 149.87, 149.84, 149.73, 149.72, 149.30, 149.21, 149.09, 149.05, 148.80, 148.72, 147.44, 147.16, 147.11, 146.94, 146.81, 146.20, 146.16, 146.00, 145.95, 145.87, 145.52, 145.47, 143.47, 143.44, 143.41, 143.39, 143.17, 143.12, 142.88, 140.98, 140.34, 140.21, 140.06, 138.09, 137.33, 135.30, 133.76, 133.75, 133.63, 133.04, 131.20, 130.82, 127.64, 127.58, 127.18, 127.05, 119.06, 118.86, 115.43, 74.31, 73.32, 73.01, 72.74, 70.55, 64.71, 63.83, 62.06, 61.85, 61.67, 61.57, 61.45, 14.61, 14.48, 14.28, 14.09. FT-IR ν/cm^−1^ (KBr) 2963, 2922, 2834, 1731, 1622, 1501, 1428, 1400, 1262, 1180, 1095, 1022, 804, 534. HRMS (MALDI-TOF) *m*/*z* calcd for C_90_H_22_N_2_O_4_ [M^−^.] 1194.1585, found 1194.1589.

#### 3.2.3. Synthesis of **P-B**

A mixture of **BOD** [31,32] (20.1 mg, 0.06 mmol), 1,6,7,12-tetra-tert-butylphenoxyperylene-3,4,9,10-tetracarboxylic dianhydride **3** (142.7 mg, 0.14 mmol), and imidazole (138.3 mg, 2.03 mmol) was stirred under an N_2_ atmosphere at 80 °C in CHCl_3_ (15 mL) for 13 h (monitored by TLC). Aniline (50 μL, 0.55 mmol) was then added, and the reaction mixture was allowed to react at 80 °C for 9 h. The mixture was cooled to room temperature and washed with water. The aqueous phase was extracted with CHCl_3_, and the combined organic phase was dried over anhydrous Na_2_SO_4_. After the solvent was removed, the residue was purified by column chromatography on silica gel with toluene as the eluent to give **PDI** [30] (37.9 mg, 41%) and **B-P** (25.4 mg, 31%). **B-P**: ^1^H NMR (300 MHz, CDCl_3_) δ 8.29 (s, 2H), 8.24 (s, 2H), 7.53–7.38 (m, 7H), 7.23 (d, *J* = 8.7 Hz, 10H), 6.85 (dd, *J* = 8.7, 1.4 Hz, 8H), 6.00 (s, 2H), 2.55 (s, 6H), 1.50 (s, 6H), 1.28 (s, 18H), 1.26 (s, 18H); ^13^C NMR (75 MHz, CDCl_3_ with Cr(acac)_3_ as relaxation reagent) δ 163.67 (*C*=O), 163.64 (*C*=O), 156.47, 156.28, 155.94, 152.88, 152.87, 147.67, 143.37, 140.67, 136.16, 135.60, 135.29, 133.39, 133.27, 131.44, 129.82, 129.40, 129.14, 128.83, 128.63, 126.90, 126.84, 122.83, 122.39, 121.63, 121.16, 120.69, 120.23, 120.19, 119.93, 119.85, 119.59, 34.52, 31.56, 14.73, 14.65. FT-IR ν/cm^−1^ (KBr) 2962, 1709, 1675, 1589, 1545, 1506, 1406, 1340, 1317, 1284, 1198, 1085, 982, 879, 834, 751. MALDI-TOF-MS *m*/*z* calcd for C_89_H_80_BF_2_N_4_O_8_ [M^−^.] 1380.5959, found 1380.6159.

#### 3.2.4. Synthesis of **C_70_-P-B**

Compound **3** (167.7 mg, 0.17 mmol), **BOD** [31,32] (25.8 mg, 0.07 mmol) and imidazole (108.5 mg, 1.61 mmol) were dissolved in 15 mL of chloroform and stirred for 30 min under an N_2_ atmosphere. The flask was equipped with an airproof stopper and stirred at 80 °C for 15 h monitored by TLC. Upon completion of the reaction, the reaction mixture was carefully quenched by water (50 mL). The mixture was transferred to a 250 mL separatory funnel. The aqueous layer was extracted with dichloromethane (2 × 50 mL). The combined organic layer was washed with saturated brine (200 mL), dried with anhydrous sodium sulfate, filtered, and concentrated. The residue was purified by TLC using CH_2_Cl_2_/PE (5:2) as an eluent to give monoanhydride as a crude product (43.6 mg, 50%). Without characterization, the crude monoanhydride was used directly. The crude monoanhydride, compound **2** (47.8 mg, 0.04 mmol), and imidazole (141.4 mg, 2.08 mmol) were dissolved in 6.5 mL of chloroform and stirred for 30 h under N_2_ atmosphere at 80 °C. Upon completion of the reaction, the reaction mixture was carefully quenched by water (50 mL). The mixture was transferred to a 250 mL separatory funnel and extracted with dichloromethane (2 × 50 mL). The combined organic layer was washed with saturated brine (200 mL), dried with anhydrous sodium sulfate, filtered and concentrated. The residue was purified on a silica gel column using CH_2_Cl_2_/PE/CS_2_ (5:1:1) as eluent to give **C_70_-P-B** as a reddish brown solid (43.4 mg, 25%). ^1^H NMR (400 MHz, CDCl_3_) δ 8.25–8.198 (m, phenyl ring H), 7.73–7.52 (m, phenyl ring H), 7.46–7.38 (m, phenyl ring H), 7.26 (dd, *J* = 8.8, 3.0 Hz, phenyl ring H), 7.16 (d, *J* = 8.6 Hz, phenyl ring H), 7.01 (d, *J* = 8.7 Hz, phenyl ring H), 6.86 (dd, *J* = 8.6, 4.3 Hz, phenyl ring H), 6.06–5.32 (s, protons of C*H*CO_2_ and pyrrole ring), 4.48–4.43 (m, OC*H*_2_CH_3_), 4.36–4.29 (m, OC*H*_2_CH_3_), 4.16–4.02 (m, OC*H*_2_CH_3_), 3.96–3.85 (m, OC*H*_2_CH_3_), 3.37–3.52 (m, OC*H*_2_CH_3_), 2.64 (s, pyrrole ring C*H*_3_), 2.56 (s, pyrrole ring C*H*_3_), 2.06 (s, pyrrole ring C*H*_3_), 1.52 (s, pyrrole ring C*H*_3_), 1.43 (t, *J* = 7.0 Hz, OCH_2_C*H*_3_), 1.329 (s, C(C*H*_3_)_3_), 1.321 (s, C(C*H*_3_)_3_), 1.313 (s, C(C*H*_3_)_3_), 1.07 (t, *J* = 7.1 Hz, OCH_2_C*H*_3_), 1.01 (t, *J* = 7.4 Hz, OCH_2_C*H*_3_), 0.94–0.88 (m, OCH_2_C*H*_3_); ^13^C NMR (100 MHz, CS_2_/CDCl_3_ with Cr(acac)_3_ as relaxation reagent) δ 169.53, 169.44, 168.97, 163.15, 163.01, 157.42, 156.18, 156.08, 155.81, 155.66, 155.08, 154.87, 154.40, 152.77, 152.70, 151.33, 151.25, 151.01, 150.92, 150.74, 150.60, 150.39, 150.30, 150.18, 149.80, 149.75, 149.65, 149.61, 149.23, 149.10, 149.02, 148.98, 148.69, 148.64, 148.61, 147.36, 147.34, 147.30, 147.29, 147.09, 147.02, 146.97, 146.86, 146.72, 146.07, 146.04, 145.93, 145.82, 145.75, 144.15, 143.42, 143.39, 143.33, 143.21, 143.12, 143.07, 140.85, 140.33, 140.22, 140.18, 139.98, 137.98, 135.88, 135.34, 133.87, 133.63, 133.54, 133.23, 133.12, 132.94, 132.34, 132.31, 132.05, 131.67, 131.51, 131.29, 131.26, 131.13, 130.73, 129.68, 128.90, 128.83, 128.78, 128.13, 128.01, 127.45, 126.73, 126.69, 122.83, 122.32, 121.52, 120.97, 120.61, 120.12, 119.73, 119.43, 119.39, 118.97, 74.23, 74.19, 72.87, 70.88, 70.47, 70.39, 65.40, 64.64, 63.77, 62.10, 61.90, 61.69, 61.51, 60.94, 60.33, 34.20, 31.42, 30.74, 29.96, 14.62, 14.60, 14.46, 14.27, 14.07, 13.93. FT-IR ν/cm^−1^ (KBr) 2959, 2924, 2852, 1708, 1673, 1592, 1504, 1402, 1339, 1283, 1174, 1090, 796, 546. HRMS (MALDI-TOF) *m/z* calcd for C_173_H_94_BF_2_N_5_O_12_ [M^−^.] 2482.7001, found 2482.7023.

### 3.3. Photooxidation Experiment

The solutions of sensitizers **C_70_-P-B**, **C_70_-Boc**, **P-B**, MB in a concentration of 2.0 × 10^−5^ mol L^−1^ and DHN in a concentration of 2.0 × 10^−4^ mol L^−1^ in CH_2_Cl_2_/MeOH (9:1, *v*/*v*) were mixed in a volume ratio of 1:1. O_2_ was then bubbled through the mixtures for 10 min, and a xenon lamp (0.17 mW/cm^2^, using 0.72 M NaNO_2_ aqueous solution as a cutoff filter) was used as a broadband light source to irradiate the mixtures. The spectral responses of the mixtures were monitored by a UV-vis spectrophotometer at intervals of 5 min.

The singlet oxygen quantum yield (Φ_∆_) was calculated by using Equation (1)

(1) $$\Phi_{\Delta }\left(x\right)={\;\Phi }_{\Delta }\left(\mathrm{std}\right)\frac{k_{\mathrm{obs}}\left(x\right)\cdot I\left(\mathrm{std}\right)}{k_{\mathrm{obs}}\left(\mathrm{std}\right)\cdot I\left(x\right)}\;$$

In this equation, Φ_∆_(x) and Φ_∆_(std) are the singlet oxygen generation quantum yield of the photosensitizers and MB. k_obs_(x) and k_obs_(std) were the absolute value of the slopes of ln[(A − A’)/A_0_] versus the irradiation time for the photooxidation of DHN by photosensitizers and MB, respectively. I(x) and I(std) were the total light intensities absorbed by photosensitizers and MB, respectively.

### 3.4. Photostability Experiment

The photostability of **C_70_-P-B** was measured in CH_2_Cl_2_/MeOH (9:1, *v*/*v*, 1.0 × 10^−5^ mol L^−1^) and irradiated continuously for 1 h using a xenon lamp (0.20 mW/cm^2^). A UV-vis spectrophotometer was then used to record the spectral responses of **C_70_-P-B** at 0 h and 1 h, respectively.

### 3.5. Measurement of Photophysical Properties

UV-Vis absorption and steady-state fluorescence were measured using an absorption spectrometer (UV-1800, Mapada, Shanghai, China) and a fluorescence spectrophotometer (FP8500, JASCO, Tokyo, Japan) at room-temperature. The fluorescence lifetimes of all the compounds were recorded via a time-correlated single photon counting (TCSPC) apparatus at room temperature, and a pulsed laser at 510 nm was used as the excitation source. The nanosecond transient absorption spectra were recorded with a home-built laser flash photolysis system. The pulsed excitation light was from the second harmonic 532 nm of a Q-Switched Nd:YAG laser (Dawa-100, Beamtech, Beijing, China) with a pulse duration of 8 ns and a 10-Hz repetition rate, and was intersected by a white light from a 500W Xenon lamp in a 10 mm × 10 mm quartz cuvette. In experiments, the pulse laser energy was set as ca.10 mJ/pulse to achieve a better signal-to-noise ratio. A monochromator equipped with a photomultiplier was used to record the transient absorption spectra within the wavelength range of 400–750 nm, with a spectral resolution of less than 1 nm. A kinetic curve of intermediate length was averaged by multi-shots and recorded with an oscilloscope (TDS3052B, Tektronix, Beaverton, OR, USA). All of the solutions were deoxygenated by purging with high purity argon (99.99%) for about 20 min prior to the measurements.

## 4. Conclusions

In conclusion, a panchromatic light-absorbing **C_70_-P-B** triad with a cascade of energy transfer has been synthesized. The photophysical processes of the triad were investigated using steady-state UV-visible absorption and fluorescence spectroscopy, time-resolved fluorescence spectroscopy, nanosecond time-resolved transient absorption spectroscopy, and theoretical calculations. After the efficient cascade of singlet excited energy transfer from BODIPY to perylene and then to C_70_ and the ISC process, the backward triplet excited state energy transfer from C_70_ to perylene moiety occurred to populate the ^3^perylene*. The nanosecond time-resolved transient absorption results show that the triplet excited states of **C_70_-P-B** are distributed on both C_70_ and the perylene moiety. The bi-exponential decay processes are found in **C_70_-P-B**. The shorter lifetime of the triplet state is 23 μs, and the longer one is 175 μs. For the synergistic effect of the antennas and C_70_, **C_70_-P-B** exhibits excellent photooxidation capacity. The photooxidation rate constant of **C_70_-P-B** is 3.70 times as that of **C_70_-Boc** and 1.58 times as that of MB, respectively. The yield of singlet oxygen of **C_70_-P-B** is as high as 0.82. The results indicate that C_70_-antennas that are panchromatic light-absorbing and have a cascade of energy transfer are useful structure motifs for generating high yields of singlet oxygen.

## Figures and Tables

**Table 1:** The photophysical properties of the photosensitizers.

Photosensitizers	λ_abs_ ^a^	λ_F_ ^b^	τ_F_ ^c^	τ_T_ ^d^	k_obs_ ^e^/min^−1^	υ_i_ ^f^	Φ_∆_ ^g^
**C_70_-P-B**	306, 399, 503, 575	607	3.7	175, 23	83.3	8.33	0.82
**C_70_-B** **oc**	398, 465, 535	-	-	42	22.5	2.25	0.81 ^h^
**B-P**	503, 575	607	5.7	-	3.7	0.37	-
MB	-	-	-	-	52.7	5.27	0.57

^a^ c = 1.0 × 10^−5^ M, in nm. ^b^ Fluorescence emission maximum, in nm. ^c^ Fluorescence lifetime, in ns. ^d^ Triplet state lifetime, in μs. ^e^ In DCM/MeOH (*v*/*v*) = 9/1. c = 10^−5^ M. The rate constant k_obs_ was calculated by the formula: ln[(A − A′)/A_0_] = −k_obs_t. In 10^−3^ min^−1^. ^f^ Initial consumption rate of DHN, υ_i_ = k_obs_[DHN]. In 10^−6^ M min^−1^. ^g^ Quantum yield of singlet oxygen (^1^O_2_), with MB as standard (Φ_∆_ = 0.57 in CH_2_Cl_2_). ^h^ Literature values [23].

## Data Availability

The data presented in this study are available in the Supplementary Materials.

## Supplementary Materials

The following supporting information can be downloaded at: https://www.mdpi.com/article/10.3390/molecules28083534/s1, The nanosecond time-resolved transient absorption decay curve of **C_70_-P-B** at 590 nm (Supplementary Figure S1), computational details (Supplementary Figure S2), the spectral response of DHN with **MB** as the sensitizer (Supplementary Figure S3), the photostability of **C_70_-P-B** (Supplementary Figure S4), ^1^H NMR, ^13^C NMR spectra, and the high resolution mass spectra (Supplementary Figures S5–S16) are also provided.

## Sample Availability

Samples of the compounds **C_70_-P-B, P-B, and C_70_-Boc** are available from the authors.
