# Supplementary material for: Panchromatic Light-Absorbing [70]Fullerene-Perylene-BODIPY Triad with Cascade of Energy Transfer as an Efficient Singlet Oxygen Sensitizer
Source: Molecules. 2023 Apr 17;28(8):3534. doi: 10.3390/molecules28083534 (PMC10144093; doi:10.3390/molecules28083534)
Supplement: Supplementary file 1 [file molecules-28-03534-s001.zip › molecules-2321884-supplementary.pdf]

Supplementary Materials

Panchromatic Light-Absorbing [70]Fullerene-Perylene-BODIPY Triad with Cascade of Energy Transfer as an Efficient Singlet Oxygen Sensitizer

Lifeng Dou [1,†], Yuanming Li [2,†], Lei Dong [1], Shuao Zhang [1], Yuanqi Wu [1], Yu Gong [1], Wei Yang [1], Hongdian Lu [1], Sane Zhu [1,3,*] and Xiaoguo Zhou [2,*]

1 School of Energy, Materials and Chemical Engineering, Hefei University, Hefei 230601, China; dou_lifeng2010@126.com (L.D.); 15955184794@139.com (L.D.); cloudrrrrrr@126.com (S.Z.); 13329282558@163.com (Y.W.); huayu202035@163.com (Y.G.); weyang@ustc.edu.cn (W.Y.); luhdo@hfuu.edu.cn (H.L.)

2 Hefei National Laboratory for Physical Sciences at the Microscale, Department of Chemical Physics, University of Science and Technology of China, Hefei 230026, China; liym@mail.ustc.edu.cn

3 State Key Laboratory of Fire Science, University of Science and Technology of China, Hefei 230026, China

* Correspondence: zhuse@hfuu.edu.cn (S.Z.); xzhou@ustc.edu.cn (X.Z.); Tel.: +86-551-62158394 (S.Z.); +86-551-63600031 (X.Z.)

† These authors contributed equally to this work.

Contents

1. Nanosecond time-resolved transient absorption decay curve of C_{70}-P-B at 590 nm S2

2. Computational details S2-12

3. The spectral response of DHN with MB as the sensitizer S13

4. The photostability of C_{70}-P-B S13

5. ^{1}H NMR, ^{13}C NMR and high resolution mass spectra S14-22

1. Nanosecond time-resolved transient absorption decay curve of C_{70}-P-B at 590 nm.

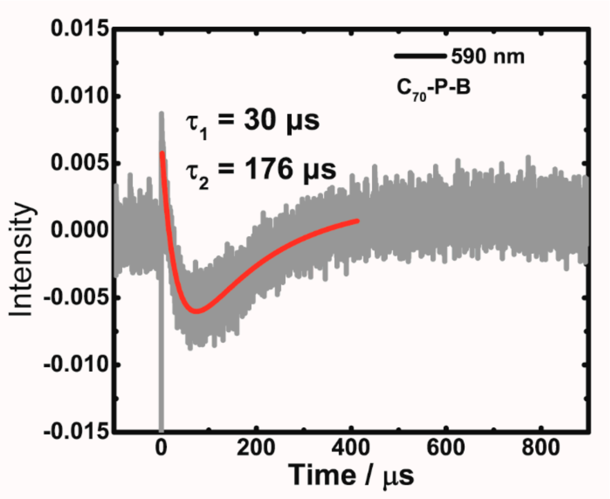

Figure S1. Nanosecond time-resolved transient absorption decay curve of **C_{70}-P-B** at 590 nm. Excited at 532 nm with a pulse laser (10 ns, 8 mJ/pulse) in deaerated toluene at room temperature.

2. Computational details

The molecular structure optimization and frontier molecular orbitals calculation involved in the triplet excited states were performed at the DFT//B3LYP/6-31G(d) and TDDFT//B3LYP/6-31G(d)with the Gaussian 09 package using toluene as the solvent.

3.1 The optimized structures of C_{70}-P-B

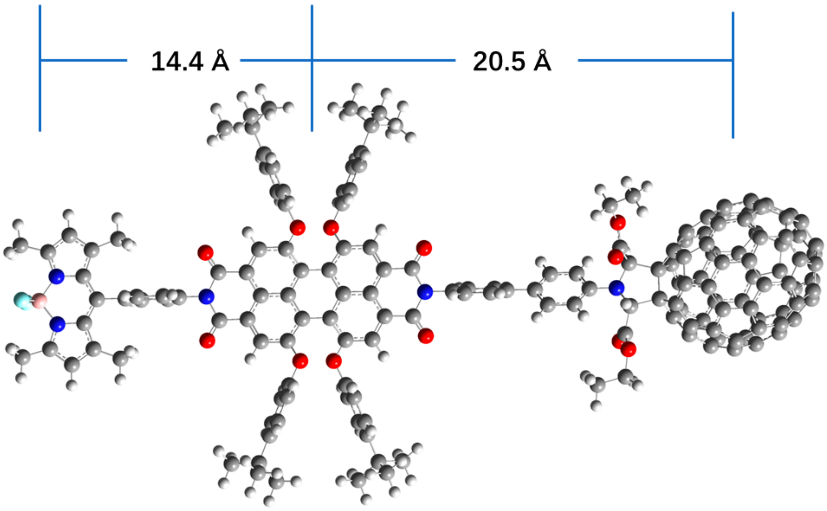

Figure S2. The optimized structure of **C_{70}-P-B**, the distance between BODIPY and perylene as well as that between perylene and C_{70} moieties.

3.2 Cartesian coordinate and energy of C_{70}-P-B

0 1

C	16.02119200	-3.69143200	1.36006600
C	15.04017200	-3.28725000	2.34742000
C	17.28499600	-3.09903100	1.34211400
C	15.35781300	-2.30814400	3.28165600
C	14.37964200	-1.29771500	3.62892500
C	13.06264400	-1.33160600	3.09671300
C	12.37743200	-0.08969400	3.01985500
C	17.19353100	-2.94384100	-1.11658300
C	15.87943900	-3.55186300	-1.09105100
C	17.88481700	-2.71625600	0.07589700
C	15.30597400	-3.92056500	0.12057900
C	13.89820100	-3.67589500	0.35126900
C	13.06455900	-3.14650800	-0.66792700
C	11.90545200	-2.44083700	-0.23587400
C	12.73245300	-2.35670600	2.10913900
C	13.73412200	-3.28539800	1.72316000
C	11.74131800	-2.05028700	1.13281000
C	18.56862800	-0.49057700	-0.73102600
C	17.84122100	-0.72857300	-1.95913100
C	18.58996700	-1.46342300	0.27364000
C	17.16935700	-1.92877600	-2.14961800
C	15.85766900	-1.92912600	-2.76717800
C	15.27188900	-0.73440600	-3.26429300
C	13.85082400	-0.70724500	-3.32386800
C	13.66446400	-2.76392100	-1.94431900
C	15.06501400	-2.93118600	-2.11569500
C	13.05802300	-1.70739300	-2.67485300

C	18.24499100	0.28027000	1.97901400
C	18.22084500	1.28123900	0.93375100
C	18.42470100	-1.06923100	1.65919000
C	18.37954700	0.90397700	-0.39275300
C	17.56427600	1.52509700	-1.42011800
C	16.66469800	2.58104000	-1.11789000
C	15.56495700	2.74356000	-2.00548900
C	15.98785200	0.53619300	-3.07191100
C	17.23044700	0.51676200	-2.38382400
C	15.23138000	1.73433800	-2.96798400
C	16.67005500	-1.69532300	3.27293700
C	16.48416400	-0.29506100	3.59363200
C	17.61728500	-2.07800100	2.32016200
C	17.25440800	0.67038200	2.95906500
C	16.64408600	1.91431000	2.52967800
C	15.28493100	2.21066200	2.81716400
C	14.64529200	3.13087200	1.94380800
C	16.49704900	2.98115500	0.28807800
C	17.24201500	2.29411400	1.28245600
C	15.24261600	3.51251900	0.69721000
C	14.46709700	1.18342000	3.48072300
C	15.07207100	-0.05613700	3.81892700
C	13.07146300	1.14888500	3.20696900
C	12.90639200	3.52480300	0.42109100
C	13.20575900	3.13389600	1.76847300
C	12.43492700	2.15002600	2.38084300
C	11.84854000	2.90194800	-0.25856300
C	13.23021400	2.75524700	-2.29446300
C	14.32984300	3.36772700	-1.57689300

C	14.17158800	3.74432100	-0.25032200
C	12.01241500	2.51284000	-1.64168600
C	11.84103600	0.31544900	-2.65212100
C	13.11201100	0.53554500	-3.30273900
C	13.79105000	1.73937700	-3.13897700
C	11.28609500	1.29242900	-1.85169800
C	10.36464400	-0.55501400	-0.20878300
C	11.24312700	-1.47335900	-1.06049200
C	11.81696600	-1.09696700	-2.24729200
C	10.38108800	0.99346100	-0.65178000
C	11.29916600	0.13163000	2.07925100
C	10.97688800	-0.83684800	1.16595500
C	11.02450700	1.92104100	0.39086800
C	11.32663500	1.54489800	1.68134100
C	8.86211000	1.31411600	-0.93740800
H	8.68893800	1.21067400	-2.01543100
C	8.83913100	-0.94322700	-0.23062800
H	8.60857700	-1.57115500	0.63088000
N	8.13817900	0.32963400	-0.15380500
C	6.72051400	0.30755100	-0.16046200
C	6.03910500	-0.52486100	0.74465100
C	5.95688400	1.10244000	-1.02906700
C	4.65044700	-0.55792500	0.77480700
H	6.59857500	-1.14199700	1.44053300
C	4.56498300	1.07263000	-0.98056300
H	6.43854500	1.74986100	-1.75362400
C	3.87665800	0.24212000	-0.08397800
H	4.15774700	-1.22900900	1.47254000
H	4.00595900	1.72185500	-1.64833800

C	8.50874300	2.74582500	-0.52106200
O	8.14812100	3.08458100	0.58275100
O	8.69929800	3.57571700	-1.56381500
C	8.47381700	-1.73353400	-1.50151500
O	8.27861000	-1.22647100	-2.58731100
O	8.39790300	-3.04649800	-1.23650300
C	8.47996900	4.99432900	-1.32495900
C	7.02172700	5.36542400	-1.53932700
H	9.13363600	5.48940600	-2.04597100
H	8.81011700	5.23066700	-0.31124700
H	6.89633800	6.44686800	-1.41475100
H	6.69479600	5.09629300	-2.54897900
H	6.38084200	4.85984000	-0.81143700
C	8.07678400	-3.93345100	-2.34612100
C	9.33209400	-4.36084200	-3.08875400
H	7.37250600	-3.42079100	-3.00441600
H	7.57966200	-4.78170600	-1.87126900
H	9.06729900	-5.08523900	-3.86709000
H	9.81383500	-3.50365500	-3.56741600
H	10.04700000	-4.83340700	-2.40771000
C	2.39393600	0.21076900	-0.04046700
C	1.70875600	0.06562900	1.17851300
C	1.63114000	0.32537300	-1.21561700
C	0.31769100	0.03515300	1.22346300
H	2.27024900	0.00207800	2.10578200
C	0.23944200	0.29713400	-1.17647000
H	2.13258600	0.41276500	-2.17489700
C	-0.41659500	0.15126400	0.04447200
H	-0.19962000	-0.06856700	2.17232900

H	-0.33838100	0.37831500	-2.09211500
N	-1.86166000	0.12206500	0.08824800
C	-2.53641000	1.35789200	0.13713400
C	-2.48786900	-1.13914100	0.12816200
C	-4.01585700	1.29891300	0.17155700
O	-1.91356000	2.41005400	0.14915900
C	-3.96868500	-1.13777700	0.16356000
O	-1.82482300	-2.16667800	0.13125600
C	-4.70318300	0.06706200	0.09612200
C	-4.72093900	2.46133900	0.39886400
C	-4.62869500	-2.32812900	0.38215300
C	-6.13046900	0.03979000	0.05938200
C	-6.12312200	2.43721900	0.45946800
H	-4.17530300	3.37748100	0.58437700
C	-6.03077400	-2.35829100	0.44205400
H	-4.04868100	-3.22421300	0.56127600
C	-6.85905300	1.28001800	0.10406300
C	-6.81063700	-1.22780500	0.09448900
O	-6.80585400	3.49617300	0.98420200
O	-6.67299800	-3.44605100	0.95912200
C	-8.31377400	1.25246400	-0.05490900
C	-8.26525900	-1.25529100	-0.06586800
C	-6.23011800	4.76184300	1.06356500
C	-6.04936700	-4.68888900	1.03437200
C	-8.99414900	-0.01517100	-0.01889100
C	-9.09440300	2.38333700	-0.40048600
C	-9.00108500	-2.41233200	-0.42246500
C	-6.20203800	5.37666200	2.31347200
C	-5.77687600	5.43850000	-0.06520100

C	-5.56549900	-5.34201100	-0.09554300
C	-6.00408000	-5.30887600	2.28125400
C	-10.42176500	-0.04260100	-0.05081400
C	-10.49571800	2.35388200	-0.33223700
O	-8.45358000	3.47102000	-0.91962400
C	-10.40272200	-2.43676200	-0.35980600
O	-8.31830300	-3.47134600	-0.94793500
C	-5.71494400	6.67694300	2.42391600
H	-6.56178800	4.83536000	3.18308600
C	-5.28357200	6.73794400	0.06837600
H	-5.81631700	4.95991700	-1.03828500
C	-5.02444500	-6.62276300	0.03383600
H	-5.61838800	-4.86022400	-1.06640100
C	-5.46931500	-6.59062200	2.38744300
H	-6.38832500	-4.78612800	3.15174100
C	-11.15554800	1.16344300	-0.11038600
C	-11.10837500	-1.27515200	-0.12696500
H	-11.07610700	3.25070800	-0.50668100
C	-9.08346600	4.71028500	-1.00365400
H	-10.94818600	-3.35284200	-0.54638200
C	-8.89771000	-4.73498900	-1.03131700
C	-5.24137700	7.39047200	1.30901000
H	-5.70219200	7.13791200	3.40728600
H	-4.93648800	7.24218200	-0.82612000
C	-4.96405100	-7.27999100	1.27123900
H	-4.65452500	-7.10881700	-0.86156900
H	-5.44443300	-7.05616200	3.36844200
C	-12.63399200	1.16543300	-0.06202800
C	-12.58580900	-1.33503100	-0.08483200

C	-9.55771900	5.37317000	0.12920000
C	-9.14699200	5.31707100	-2.25220400
C	-9.34821600	-5.41441500	0.10148400
C	-8.93439400	-5.34580600	-2.27898600
C	-4.71050400	8.82509300	1.48232300
C	-4.38176500	-8.69509800	1.43968200
O	-13.30239800	2.18979100	-0.05531700
N	-13.25789900	-0.09782600	-0.02067700
O	-13.21386600	-2.38456000	-0.10029200
C	-10.10408300	6.64572900	-0.00894300
H	-9.48988900	4.90044700	1.10356200
C	-9.69063700	6.59931700	-2.36818500
H	-8.77018100	4.78878500	-3.12260000
C	-9.84386700	-6.70760900	-0.03581600
H	-9.30169900	-4.93781000	1.07519900
C	-9.42707200	-6.64858800	-2.39411300
H	-8.57680500	-4.80443700	-3.14945700
C	-4.24229600	9.44020900	0.14987900
C	-3.50904600	8.81554900	2.45743800
C	-5.83166600	9.72203000	2.05945600
C	-3.88158800	-9.28400600	0.10699800
C	-3.18842000	-8.64778800	2.42358300
C	-5.47340800	-9.63617000	2.00295300
C	-10.18423700	7.29344700	-1.25537800
H	-10.46706400	7.14533400	0.88439700
H	-9.72671600	7.04916300	-3.35381300
C	-9.89571600	-7.35988300	-1.28136600
H	-10.18929500	-7.21957300	0.85748900
H	-9.44329700	-7.10090800	-3.37913200

H	-3.87402200	10.45728300	0.32439800
H	-3.42462100	8.86583300	-0.30011700
H	-5.05829900	9.50512200	-0.57901700
H	-3.12420400	9.83319900	2.59610700
H	-3.78648600	8.42759900	3.44325100
H	-2.69436000	8.19352900	2.06961900
H	-6.69596900	9.75147800	1.38580400
H	-6.17909200	9.36462900	3.03462900
H	-5.46742400	10.74811100	2.19138900
H	-3.08302400	-8.67652000	-0.33375700
H	-3.47655500	-10.28760400	0.27812300
H	-4.68945500	-9.37506900	-0.62809700
H	-3.48664800	-8.27627400	3.40966100
H	-2.76773600	-9.65159100	2.55903600
H	-2.39424900	-7.99415400	2.04524500
H	-5.84031700	-9.29817900	2.97790600
H	-6.33146100	-9.69222700	1.32302300
H	-5.07309200	-10.64925300	2.13078600
C	-10.79144700	8.70486900	-1.34954000
C	-10.44747100	-8.79397700	-1.37467300
C	-12.25411800	8.67116100	-0.84566000
C	-10.79311700	9.24550100	-2.79196600
C	-9.96976200	9.67824300	-0.47061100
C	-11.91185700	-8.81583200	-0.87513600
C	-10.42368400	-9.33668900	-2.81611900
C	-9.59147500	-9.73342300	-0.49154600
H	-12.69800100	9.67257500	-0.90081100
H	-12.86343600	7.99477100	-1.45585300
H	-12.32026200	8.33451300	0.19425800

H	-11.38567000	8.61417200	-3.46386800
H	-11.23386100	10.24848700	-2.80781200
H	-9.77897800	9.32421900	-3.20015100
H	-9.96579400	9.37396700	0.58136900
H	-8.92832100	9.72763700	-0.80929800
H	-10.39345600	10.68845700	-0.52379800
H	-12.54506000	-8.16491800	-1.48870300
H	-12.31661900	-9.83376600	-0.92901100
H	-11.99394800	-8.47945200	0.16373300
H	-10.82502300	-10.35608500	-2.83158700
H	-11.03819400	-8.72997600	-3.49096100
H	-9.40596000	-9.37655000	-3.22103100
H	-8.54775900	-9.74285500	-0.82675500
H	-9.60276400	-9.42778100	0.55998100
H	-9.97541200	-10.75942700	-0.54452400
C	-14.70096300	-0.12523400	0.04560100
C	-15.44880500	-0.19164500	-1.12875100
C	-15.33594800	-0.08378400	1.28559100
C	-16.84083600	-0.21679900	-1.06112600
H	-14.94320400	-0.22374300	-2.08870600
C	-16.72842300	-0.10752200	1.35052000
H	-14.74274100	-0.03234600	2.19321800
C	-17.49244200	-0.17427400	0.17835700
H	-17.42582200	-0.26884700	-1.97471400
H	-17.22556000	-0.07421000	2.31558500
C	-18.98552100	-0.19880600	0.24905600
C	-19.69645100	1.01233400	0.23281700
C	-19.65222300	-1.43231000	0.33009300
C	-19.26149300	2.37817300	0.15460800

N	-21.09615800	1.01096500	0.29934500
C	-19.16795600	-2.78330200	0.36150700
N	-21.05119400	-1.47635600	0.39675600
C	-20.41918700	3.14850500	0.17751100
C	-17.87175700	2.93704600	0.06385400
C	-21.53065300	2.28716700	0.26658500
B	-22.00325400	-0.24763100	0.39631600
C	-20.29712200	-3.59088000	0.44563500
C	-17.75848000	-3.29642600	0.31704700
C	-21.43923100	-2.76603900	0.46574800
H	-20.47040900	4.22908400	0.13434100
H	-17.25590800	2.66176100	0.92640900
H	-17.33874100	2.58252100	-0.82454300
H	-17.91669600	4.02936800	0.01665300
C	-22.97865000	2.65114800	0.31979200
F	-22.74550800	-0.21456800	1.58004700
F	-22.86282700	-0.30629200	-0.70385600
H	-20.30903900	-4.67259000	0.48907500
H	-17.23849600	-3.00345200	-0.60106300
H	-17.15389000	-2.92224300	1.14981800
H	-17.76288600	-4.38951600	0.36774600
C	-22.87339900	-3.17643600	0.54826600
H	-23.52035000	2.20313600	-0.52004400
H	-23.44045000	2.26546500	1.23492500
H	-23.10118600	3.73647500	0.28660500
H	-23.35017700	-2.73777000	1.43129000
H	-23.42941400	-2.81435500	-0.32309700
H	-22.95712000	-4.26474900	0.59943900

E = - -8050.227826 Hartree

3. The spectral response of DHN with MB as the sensitizer.

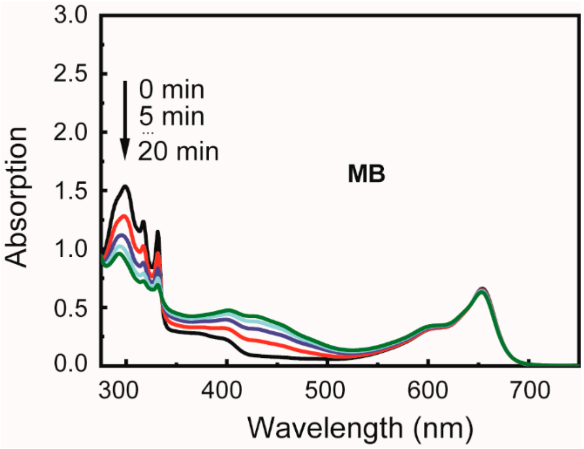

Figure S3. Absorption spectral change for the photooxidation of DHN using **MB** as the sensitizer. c[MB] = 1.0×10^{-5} mol L^{-1}, c[DHN] = 1.0×10^{-4} mol L^{-1}. In CH_2Cl_2–MeOH (9/1, v/v).

4. The photostability of C_{70}-P-B.

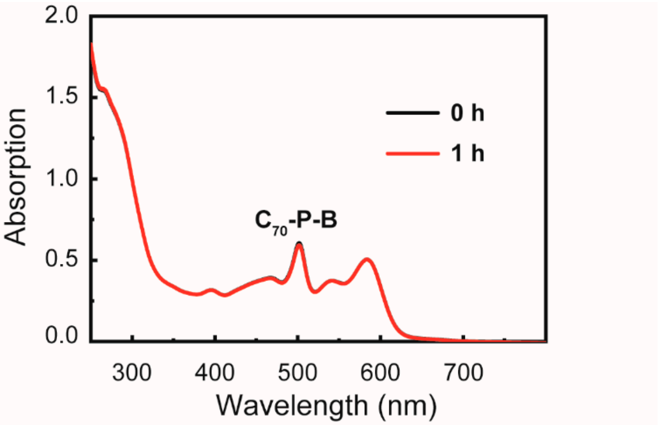

Figure S4. The stability of **C_{70}-P-B**. c = 1.0×10^{-5} mol L^{-1} in CH_2Cl_2–MeOH (9/1, v/v). After being exposed to light for 1 h, no bleaching is observed.

5. ¹H NMR, ¹³C NMR and high resolution mass spectra

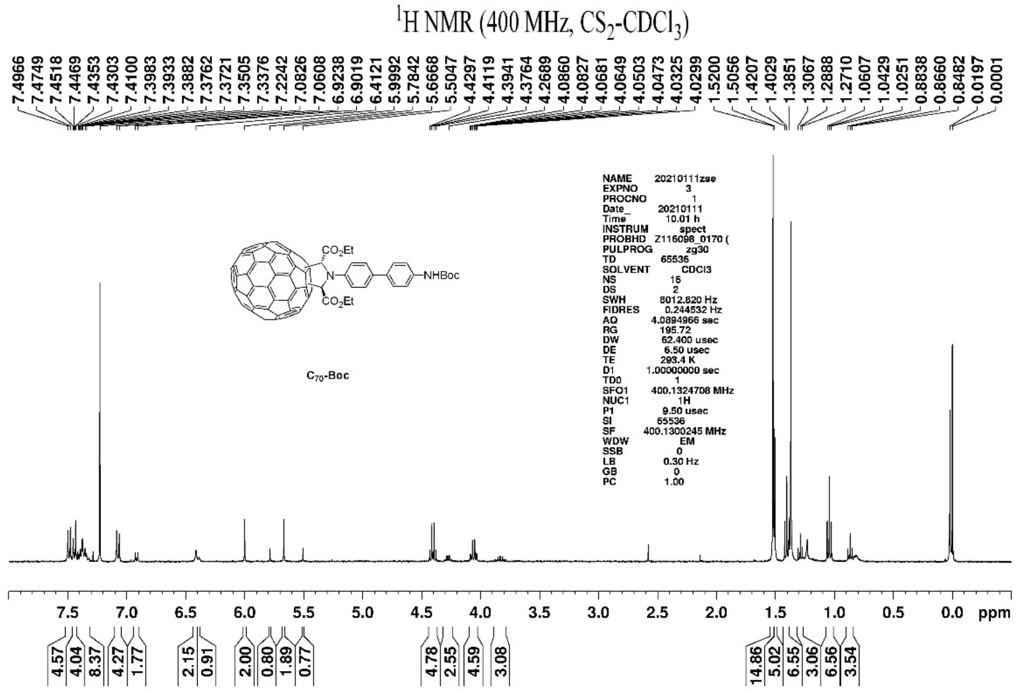

Figure S5. ¹H NMR of **C_{70}-Boc** in CS_2-$CDCl_3$ (400 MHz).

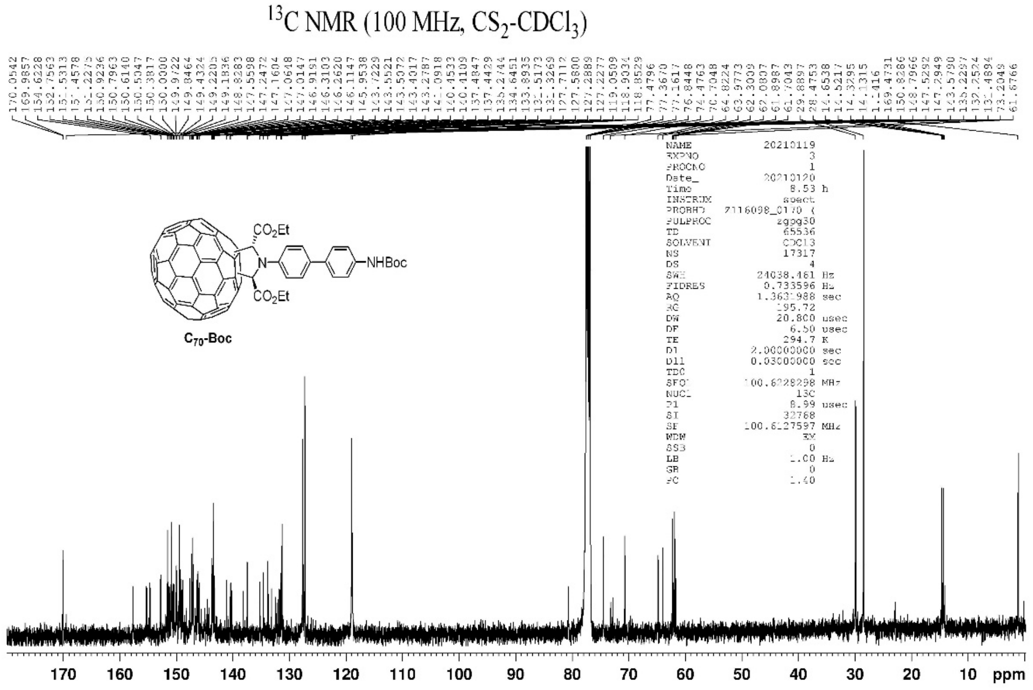

Figure S6. ¹³C NMR of **C_{70}-Boc** in CS_2-$CDCl_3$ (100 MHz).

D:\data\gc\20201112\(1)\0_H24\1\1Ref

Comment 1
Comment 2

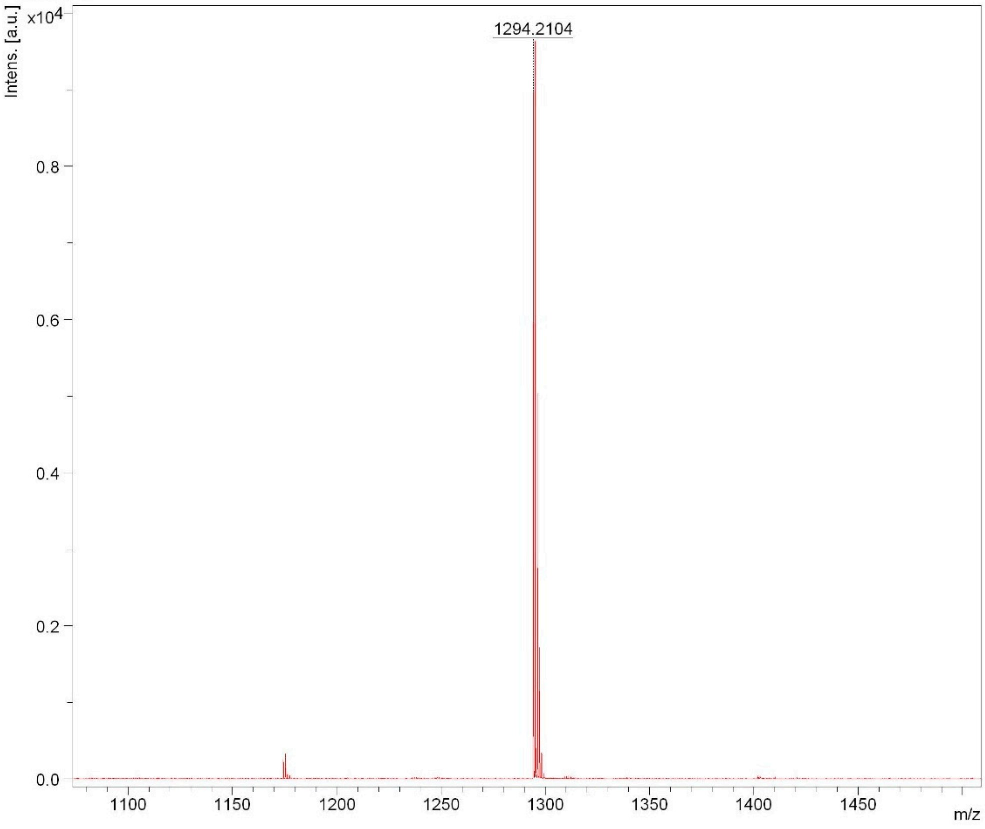

Acquisition Parameter

Date of acquisition	2020-11-13T10:00:29.174+08:00
Acquisition method name	D:\Methods\flexControlMethods\gc-RN_0-1200_Da.par
Aquisition operation mode	Reflector
Voltage polarity	NEG
Number of shots	500
Name of spectrum used for calibration	
Calibration reference list used	sample

Instrument Info

User	BDAL@CN
Instrument	FLEX-PC
Instrument type	ultraflexTOF/TOF

Bruker Daltonics flexAnalysis printed: 2020-11-13 13:59:01

Figure S7. The high resolution mass spectrum (HRMS) of **C_{70}-Boc**.

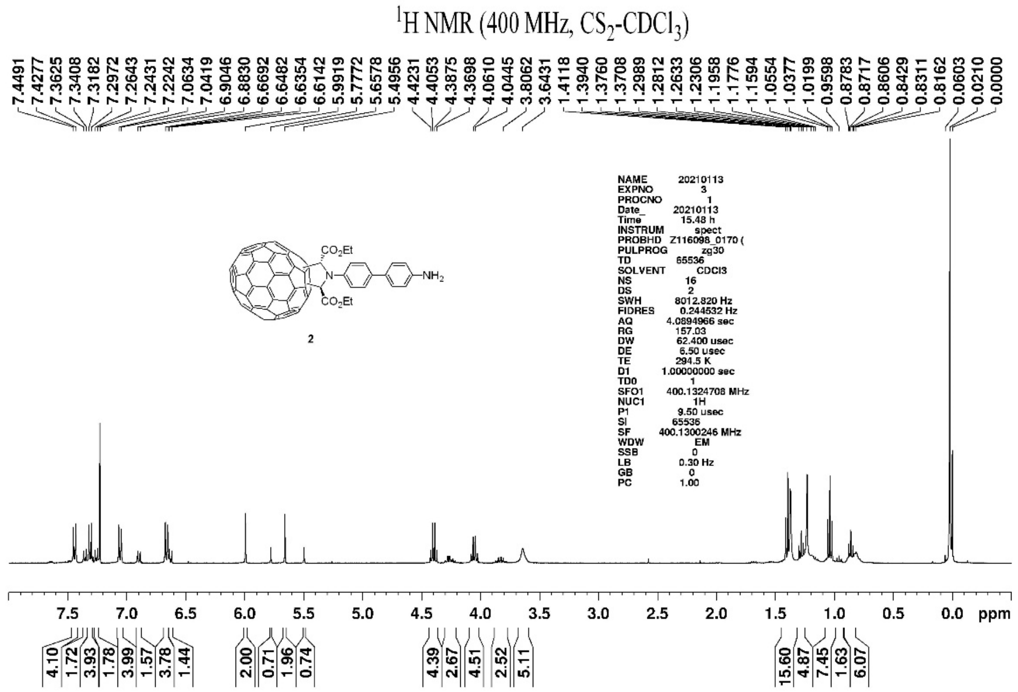

Figure S8. ^{1}H NMR of **2** in CS_2-$CDCl_3$ (400 MHz).

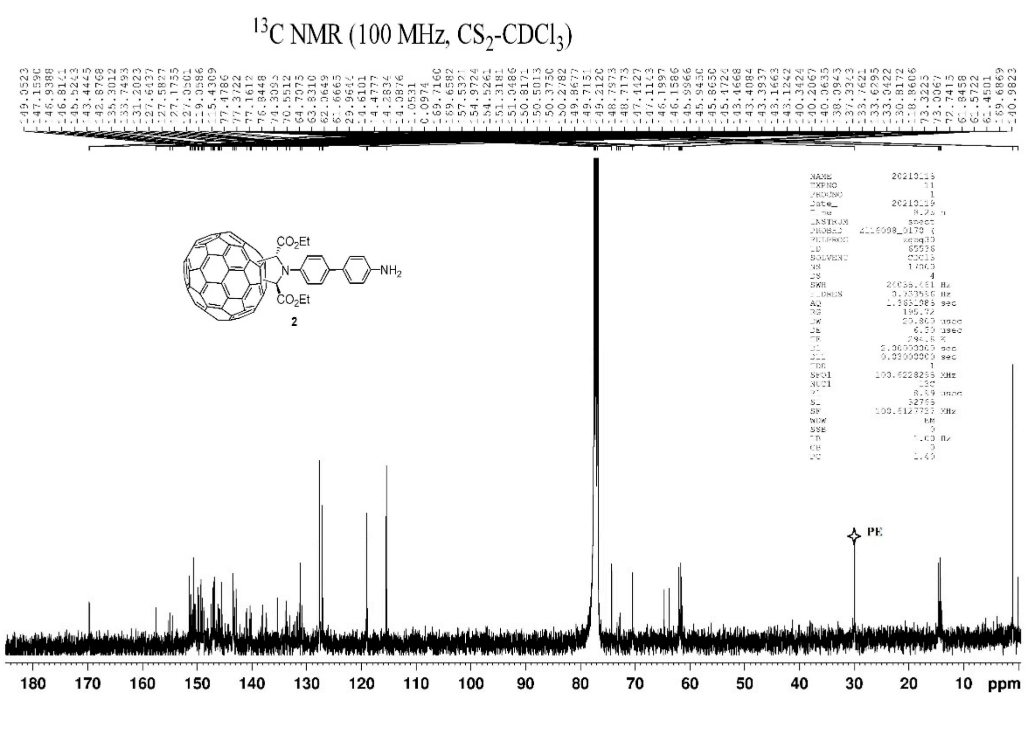

Figure S9. ^{13}C NMR of **2** in CS_2-$CDCl_3$ (100 MHz).

D:\data\gc\20210201\P-2\0_K24\1\1Ref

Comment 1

Comment 2

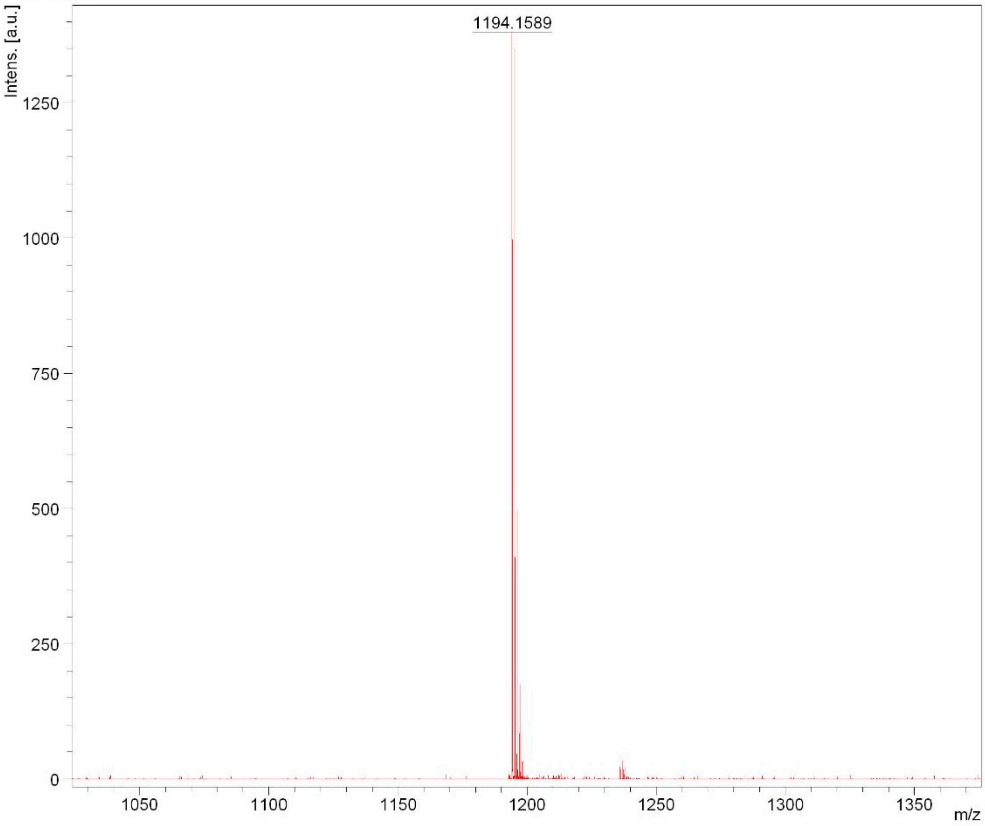

Acquisition Parameter

Date of acquisition	2021-02-01T21:25:35.281+08:00
Acquisition method name	D:\Methods\flexControlMethods\gc-RN_0-1200_Da.par
Aquisition operation mode	Reflector
Voltage polarity	NEG
Number of shots	500
Name of spectrum used for calibration	
Calibration reference list used	sample

Instrument Info

User	BDAL@CN
Instrument	FLEX-PC
Instrument type	ultraflexTOF/TOF

Bruker Daltonics flexAnalysis printed: 2021-2-1 22:12:32

Figure S10. The HRMS of **2**.

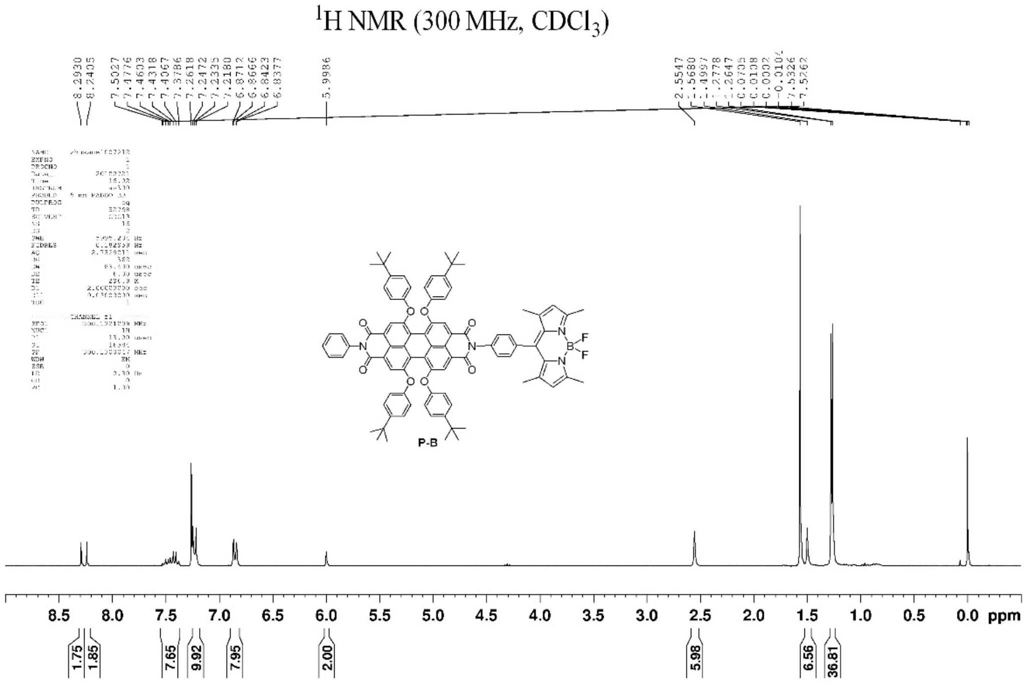

Figure S11. ^{1}H NMR of **P-B** in $CDCl_3$ (300 MHz).

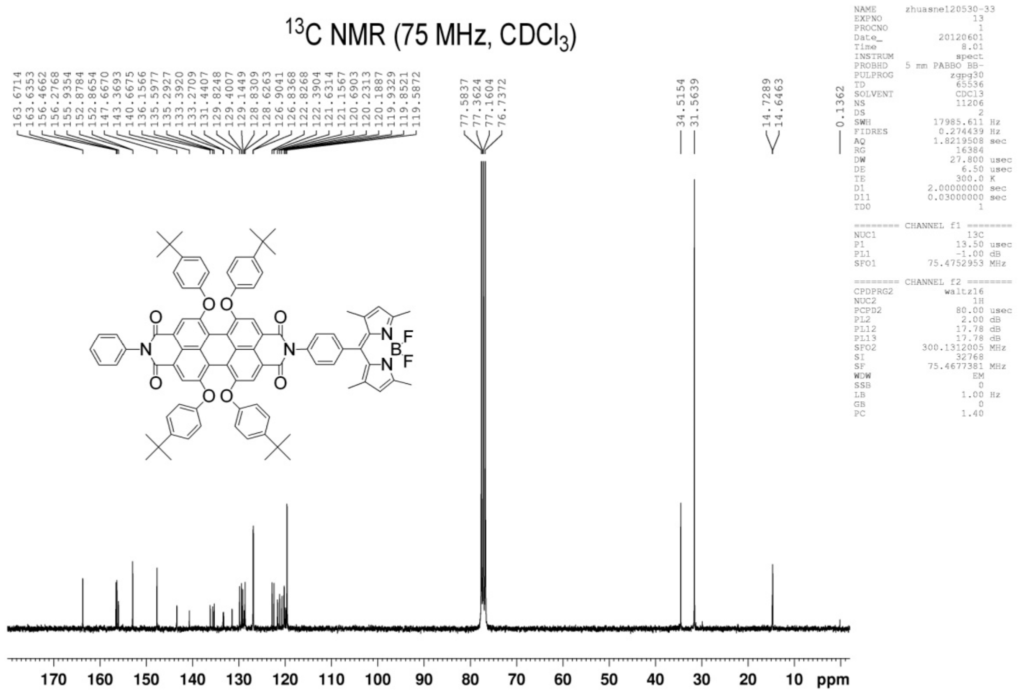

Figure S12. ^{13}C NMR of **P-B** in $CDCl_3$ (75 MHz).

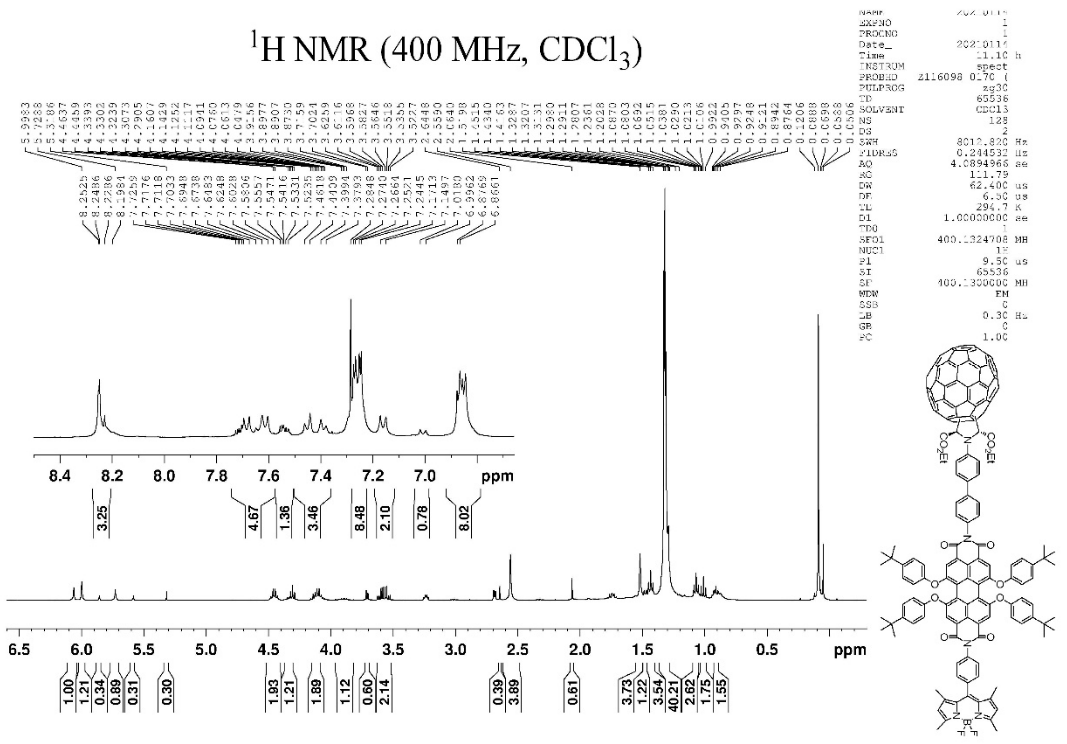

Figure S13. ^{1}H NMR of **C_{70}-P-B** in $CDCl_3$ (400 MHz).

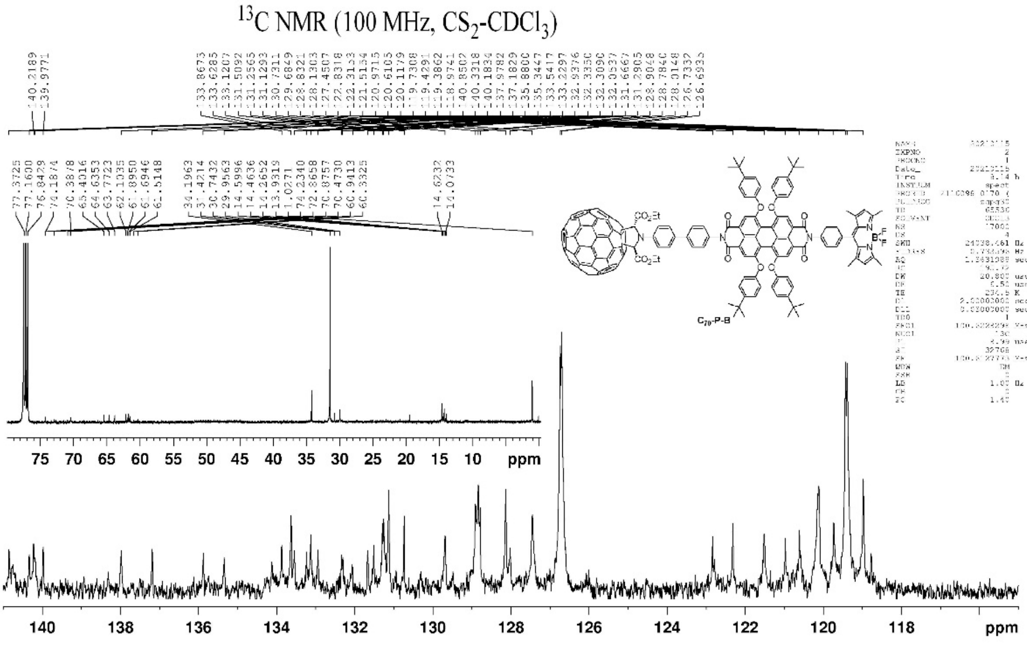

Figure S14. Expansion of the ^{13}C NMR of **C_{70}-P-B** in $CDCl_3$ (100 MHz).

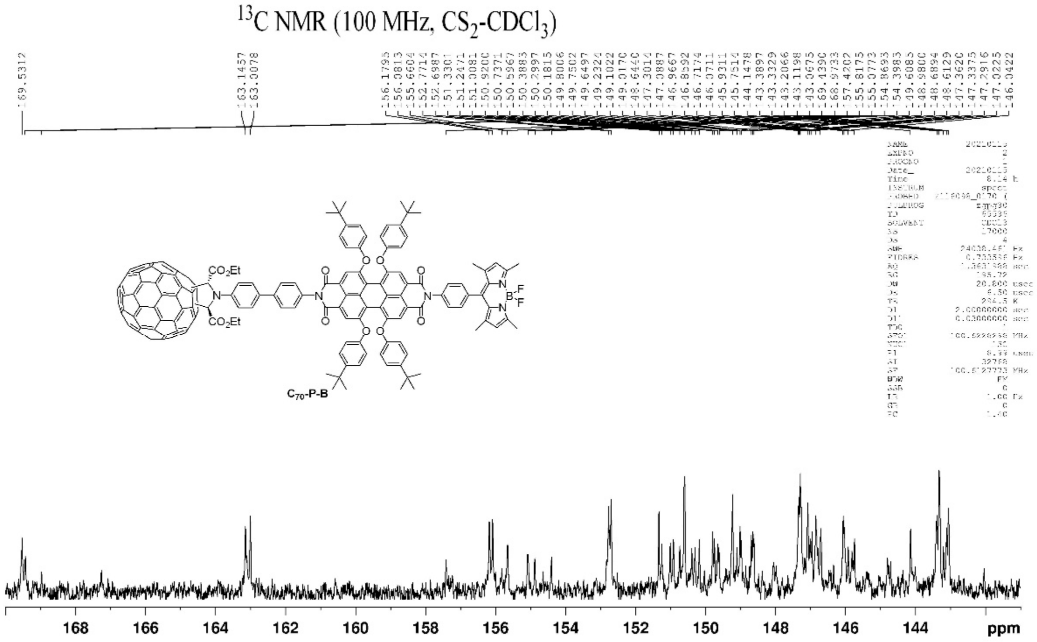

Figure S15. Expansion of the ^{13}C NMR of **C_{70}-P-B** in $CDCl_3$ (100 MHz).

D:\data\gc\20210201\P-3\0_L1\1\1Ref

Comment 1

Comment 2

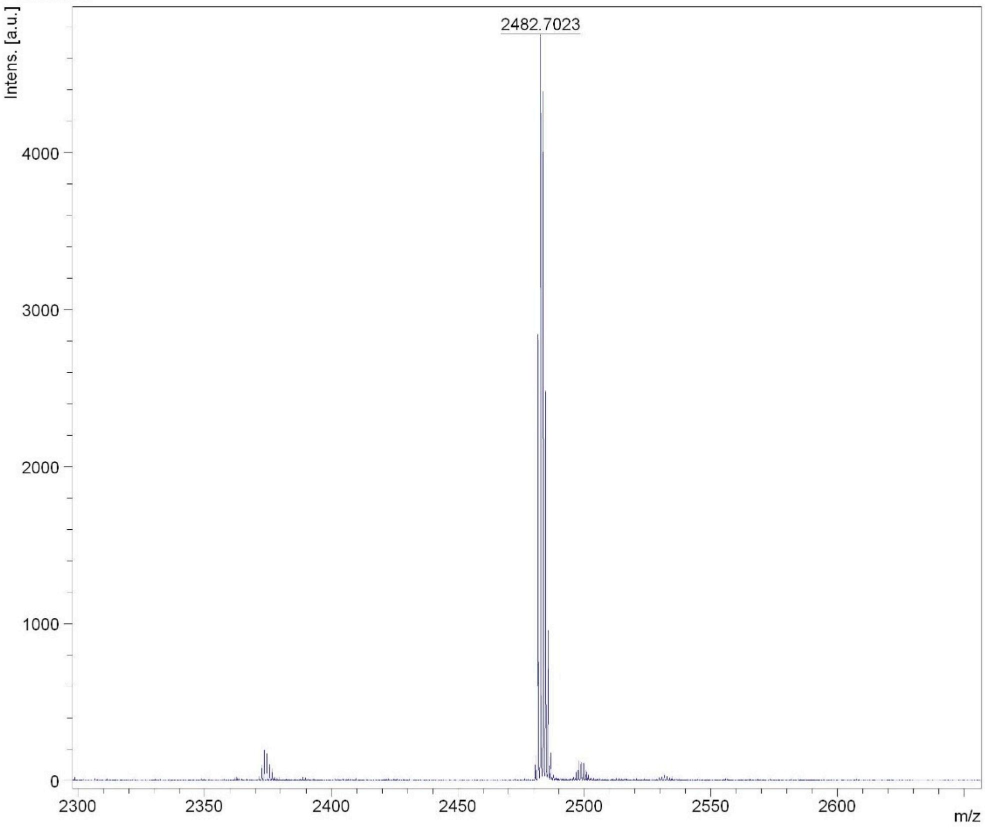

Acquisition Parameter

Date of acquisition	2021-02-02T08:56:00.781+08:00
Acquisition method name	D:\Methods\flexControlMethods\gc-RN_0-1200_Da.par
Aquisition operation mode	Reflector
Voltage polarity	NEG
Number of shots	500
Name of spectrum used for calibration	
Calibration reference list used	sample

Instrument Info

User	BDAL@CN
Instrument	FLEX-PC
Instrument type	ultraflexTOF/TOF

Bruker Daltonics flexAnalysis printed: 2021-2-2 8:57:44

Figure S16. The HRMS of **C_{70}-P-B**.
